# The Preclinical Effects and Mechanisms of Biofield Therapy on Pancreatic Cancer Cell Growth and Metastasis

**DOI:** 10.1002/cam4.71726

**Published:** 2026-04-13

**Authors:** Peiying Yang, Daoyan Wei, Sharmistha Chakraborty, Phuong Nguyen, Andrew Cusimano, Defeng Deng, Shafaqmuhammad Iqbal, Monica Nelson, Meng Cui, Jianliang Dai, Mihai Gagea, Richard Wagner, Yisheng Li, Lorenzo Cohen

**Affiliations:** ^1^ Department of Palliative, Rehabilitation, and Integrative Medicine The University of Texas MD Anderson Cancer Center Houston Texas USA; ^2^ Department of Gastroenterology and Hepatology The University of Texas MD Anderson Cancer Center Houston Texas USA; ^3^ Department of Biostatistics The University of Texas MD Anderson Cancer Center Houston Texas USA; ^4^ Department of Veterinary Medicine and Surgery The University of Texas MD Anderson Cancer Center Houston Texas USA

**Keywords:** biofield therapy, FOXM1, metastasis, pancreatic cancer

## Abstract

**Background:**

The high mortality of pancreatic ductal adenocarcinoma (PDAC) underscores the need for novel treatments. We investigated the potential role of human biofield therapy (BT) in modifying tumorigenic processes in murine and human PDAC cells through a series of in vitro and in vivo studies.

**Methods:**

Cell viability and ultrastructure changes were examined by PrestoBlue assay and Transmission Electron Microscope, respectively. Cell voltage potentials were assessed using DiBAC4 staining. Both FOXM1 CRISPR KO and Tet‐on FOXM1 overexpressing PDAC cells were used for the mechanistic studies. Antitumor efficacy was tested in orthotopic models using human PANC‐1 and mouse KPCY cells.

**Results:**

Multiple mouse and human PDAC cell lines exposed to BT (three different therapists using a standardized procedure) showed significantly reduced proliferation compared to sham control (SC) and incubator control (IC) cells across multiple studies. BT induced swollen mitochondria in PANC‐1 cells and increased G1 cell cycle arrest in PANC‐1, L3.7, and COLO357 cells. BT also downregulated key cell cycle regulators including FOXM1 in PANC‐1 and L3.7 cells (*p* < 0.05). BT led to a 36.7% reduction of cell voltage potential measured in PANC‐1 cells (*p* < 0.01), with similar effects in MiaPaCa‐2 and KPCY cells. Furthermore, BT reduced the invasiveness in PANC‐1, L3.7, and COLO357 cells, an effect influenced by FOXM1 gene expression. BT inhibited the primary tumor growth and liver metastasis of PANC‐1 and KPCY cells in orthotopic mouse models (*p* < 0.05).

**Conclusions:**

These novel findings support the potential of BT to influence growth and metastasis of pancreatic cancer cells and warrant further investigation.

## 1 Introduction

Pancreatic ductal adenocarcinoma (PDAC) is one of the most aggressive cancers and currently the third leading cause of cancer‐related death in the United States [1]. Despite improvements in surgical techniques and systemic and targeted therapies, pancreatic cancer is only modestly affected by most existing treatments, including immunotherapy [2, 3, 4], with a 5‐year overall survival rate of < 13% and average survival duration from diagnosis of < 6 months [5]. Cancer‐associated mortality in PDAC is primarily caused by early metastasis, therapeutic resistance, or recurrence; thus, more effective treatments are needed.

Complementary and alternative medicine interventions have provided unanticipated insights into disease processes and have led to efficacious therapeutic interventions becoming part of mainstream medicine [6]. For example, acupuncture, once viewed as “quackery” and a “pseudoscience,” is now part of multiple evidence‐based clinical care guidelines in oncology [7, 8]. At the same time, these approaches are slow to adopt due to limited insights into the biological and mechanistic bases for such interventions on pathophysiological processes. One such intervention is biofield therapy (BT), which the National Center for Complementary and Integrative Health classifies as an “energy therapy.” Clinical trials that have examined the effects of BTs such as therapeutic touch, healing touch, and Reiki have shown that these treatments led to improvements in subjective outcomes such as pain and anxiety and some indication of improvements in immunologic parameters [9, 10]. More rigorous, blinded studies with objective outcomes such as immune function, stress hormone, and inflammatory cytokine regulation have also documented effects of BT [11, 12, 13]. Although these findings were generally supportive, research on BT is limited by multiple methodologic challenges in conducting clinical trials.

Preclinical studies, which are less subject to experimental biases (e.g., patient blinding, placebo effects), have shown some evidence that BT modifies cellular function and tumor growth [14]. For example, Gronowicz et al. [14] showed that BT modulated DNA synthesis and human osteoblast mineralization in culture studies. They also demonstrated that BT inhibited metastasis and modulated immune responses in BALB/c mice injected with mouse breast cancer 66c14 cells [14]. We previously reported that BT led to reduced cell viability and downregulation of pAkt in non‐small cell lung cancer cells and either significantly slowed the growth of mouse Lewis lung carcinoma or led to a significantly more necrotic and apoptotic tumor phenotype [15, 16]. We also found that the antitumor effect of BT was potentially mediated by modification of the tumor microenvironment and the stemness of the tumor cells [16]. A recent small study by our group showed BT resulted in decreased invasiveness of PANC‐1 cells and less cellular uptake of Ca^2+^, with changes in Ca^2+^ and tubulin correlated with changes in the BT participants' electroencephalogram (EEG) [17]. However, whether BT can affect the development and progression of PDAC and its potential mechanisms has not been systematically studied.

We conducted a series of studies to explore the effects of BT in different in vitro and in vivo cancer models of PDAC (preregistered at https://osf.io/qtbvf/wiki/home/). In the current study we determined the effects of BT on: (1) proliferation and invasion of PDAC cells, human PDAC‐derived organoids, and their relevant molecular mechanisms; (2) visible cellular morphology change; (3) resting cell membrane voltage potential (*V*_*m*_) of cells as a mechanistic link between BT and cancer phenotype; and (4) primary tumor growth and metastasis in orthotopic pancreatic cancer mouse models. We also sought to replicate key in vitro studies with three biofield therapists, with sham personnel included in these series of experiments. The BT practitioners used the Bengston energy healing method [18, 19] (see Section 2). We hypothesized that BT would reduce the growth of both human and mouse pancreatic cancer cells, alter cell invasion and liver metastasis in orthotopic mouse models engrafted with human PANC‐1 and mouse KPCY PDAC cells, respectively.

## 2 Materials and Methods

### 2.1 Reagents and Antibodies

All procedures were performed according to the relevant guidelines, rules, and regulations of The University of Texas MD Anderson Cancer Center. PrestoBlue was purchased from Thermo Fisher Scientific (San Francisco, CA); an invasion kit and cell migration assay kit from Cell Biolabs (San Diego, CA); DiBAC4 (bis‐[1,3‐dibutylbarbituric acid] trimethine oxonol) (B438) from Thermo Fisher; and gemcitabine from MD Anderson Pharmacy. Antibodies against FOXM1 (5436), p21 (2947), N‐cadherin (13116), and E‐cadherin (14472) were purchased from Cell Signaling Technology (Danvers, MA), and anti‐actin (MAB8929) was purchased from R&D Systems (Minneapolis, MN). For running the Jess system, a 12–230 kDa Separation Module, 8 × 25 capillary cartridges (SM‐W004), Anti‐Rabbit Detection Module (DM‐001), and Anti‐Mouse NIR Detection Module (DM‐009) were purchased from ProteinSimple (San Jose, CA). Nucleus live cell staining dye Invitrogen NucBlue Live Ready Probes Reagent (Hoechst 33342, R37605) was purchased from Thermo Fisher.

### 2.2 Cell Lines and Patient‐Derived Organoids (PDO)

Human pancreatic cancer cells (PANC‐1 and MiaPaCa‐2) were purchased from the American Type Culture Collection (Manassas, VA), and the human pancreatic cancer cells COLO357 and L3.7, normal human pancreatic ductal epithelial cells HPDE‐1/E6E7, and mouse pancreatic cancer Panc02 cells were used as described previously [20]. Mouse pancreatic cancer KPCY cell line (clone 6419c5, Cat# EUP005‐FP), originally derived from the autochthonous KPC mouse model of PDAC [21], was purchased from Kerafast (Boston, MA). KPCY 6419c5 was characterized as a T‐cell low infiltration clone, which has higher metastatic potential [21]. All cancer cells were maintained in a humidified atmosphere with 5% carbon dioxide at 37°C. PANC‐1, Panc02, COLO357, and L3.7 cells were routinely cultured in either Dulbecco's modified Eagle medium with high glucose or RPMI 1640 medium (Invitrogen Corp, Grand Island, NY) containing 10% fetal bovine serum (Hyclone Laboratories Inc., Logan, UT) supplemented with 50 IU/mL penicillin, 50 μg/mL streptomycin, and 2 mM L‐glutamine from GIBCO (Invitrogen). HPDE‐1/E6E7 cells were cultured in Gibco Keratinocyte SFM (Cat# 17–005‐042) with supplements of bovine pituitary extract (25 mg/500 mL) and EGF 2.5 μg/500 mL (Invitrogen). All human cell lines were authenticated by the Cytogenetics and Cell Authentication Core at MDACC using the short tandem repeat (STR) profiling method and were free from mycoplasma contamination. The cell lines were chosen based on our previous research showing that the PANC‐1, MiaPaCa‐2, COLO357, and L3.7 cells have relatively higher FOXM1 expression compared to other cell lines including BxPC3, AsPC‐1, and MDA28 [22].

Pancreatic cancer PDOs (AM67) were procured from Dr. Anirban Maitra's laboratory at The University of Texas MD Anderson Cancer Center. The organoids were dislodged using 2 mg/mL dispase in phosphate‐buffered saline (PBS) for 15 min, followed by washing with PBS at 1400 rotations per minute for 2 min and dissociation with TrypLE for 5 min at 37°C. TrypLE was quenched with Wash Media, cells were centrifuged again for 5 min at 125× *g*, and dissociated cells were counted and resuspended in 350–700 μL (depending on cell pellet size) of Geltrex LDEV‐Free, hESC‐Qualified, Reduced Growth Factor Basement Membrane Matrix (Gibco) and plated as domes on a Nunclon Delta Surface 12‐well plate (Thermo Fisher). Matrigel was allowed to solidify for up to 45 min at 37°C, at which point approximately 1 mL of PaTOM media68 supplemented with Y‐27632 dihydrochloride (Tocris, Minneapolis, MN) was added to each well of the 12‐well plate and used throughout the entire PDO culture to ensure full submergence of organoid domes. Organoids were grown for a minimum of 7 days after seeding before being passaged for the first time.

### 2.3 BT Procedures for In Vitro Studies

Most of the experiments were conducted by one therapist (Therapist 1), and some of the cell experiments were replicated with two other therapists (indicated as Therapist 2 or Therapist 3 in the figures). Initial cell experiments explored a possible dose–response effect and examined exposures of 5, 15, and 30 min, as well as 60 and 120 min (data not shown) of BT. There was no clear indication of a dose–response effect in the majority of tests, and, depending on the cell line, the 15‐ and 30‐min exposures generally had the strongest effect and were used for most of the experiments. Unless otherwise indicated, treatments were provided for 15 min. Each study had two or three groups: (1) BT treatment group, where cells were either on the counter or treated through an incubator; (2) sham counter control (SC), where the plated cells were on the counter for the same amount of time as in the BT group and a person mimicked the movements and distance from the cells as in the BT group; and (3) for some experiments, plated cells remained in the incubator (incubator control [IC]). The temperature and CO2 levels in both control incubator and treatment incubator were similar. Key laboratory members and those who performed pathologic assessment and the RPPA analyses were blinded to group assignment when conducting the assays or evaluating the animals.

### 2.4 BT and Sham Protocol

As human subjects were involved with the experiments, the study was approved by the Institutional Review Board of The University of Texas MD Anderson Cancer Center (protocol 2020–1210). All BT and Sham participants provided informed consent before participating.

#### 2.4.1 BT Protocol

The BT protocol used was the Bengston Energy Healing Method. This involves creating images that the therapist cycles through as rapidly as possible while using a hands‐on technique. Detailed descriptions of these rapid imaging and hands‐on techniques have been described previously [18, 19, 23]. Briefly, the therapist rapidly cycles through images or symbols of future events they want to have or experience. Each event needs to be tangible enough to know when it has been achieved. The technique emphasizes speed, repetition, and nonattachment rather than belief or directed effort. While the therapist cycles through the images, they are in a seated position in front of the target receiving the treatment (petri dishes or animal cages). They are in a relaxed state with their hands approximately 6 to 24 in. from the target and may engage in slow movements with their hands but never touching the dishes/cages/animals. Our prior study [17] found EEG brain activity changes were mostly localized in occipital regions during BT, indicating increased visual processing, consistent with the visualizations of the BT technique.

Three different BT practitioners participated in the experiments: two male, aged 70 (Therapist 1) and 44 (Therapist 3), and one female aged 61 (Therapist 2). All three were trained in the Bengston Method with experience ranging from 7 years (Therapist 3) to more than 13 years (Therapist 1). Therapists 1 and 3 had other prior BT trainings. To standardize the procedure, all three therapists used the same Bengston Method. They also maintained the same distance from the targets receiving the treatments, were consistent with their body distance and the use of their hands, and followed the treatment timings as instructed by laboratory staff.

#### 2.4.2 Sham Protocol

In the experiments using sham, a sham control (SC) laboratory personnel member sat in front of the cells or cages of mice and mimicked the movements/distance from the cells/animals used by the biofield therapist. They also focused on the cells or mice in the same way as the therapists, but they did not engage in any specific visualizations. This controlled for the presence of a person and the passage of time in the same laboratory conditions as the BT cells/animals. They were not trained in the Bengston Method.

### 2.5 Cell Proliferation and Growth In Vitro

Human or mouse pancreatic cancer cells (1 × 10^4^) were seeded in 96‐well plates. After incubation for 16–24 h, cells were exposed to BT. Immediately after the treatment, cell growth was assessed by a PrestoBlue assay [24].

Human pancreatic ductal epithelial HPDE‐1/E6E7 cells (1 × 10^4^) were plated in 96‐well plates. After incubation for 16 h, cells were exposed to BT in the normal cell culture medium as described in the previous section. Immediately after the treatment, cell growth was determined by a PrestoBlue assay [24].

To assess the growth of PDOs, acid solubilized Rat tail collagen I (3 mg/mL, Corning, USA) was used. It was first neutralized with 375 μL of 10× DMEM (with Phenol red) and 1N NaOH (100 μL). Neutralized Collagen I (25 μL) was used to make underlays in 12 wells plate (NUNC, Life Technology) and was polymerized on ice for 2 h until the underlays turned cloudy and were transferred to 37°C at least 30′‐1 h before cell plating. About 500 single pancreatic organoid cells were mixed with cold neutralized collagen [25] and were plated gently over the underlays on a 37°C heating plate. Plates were incubated at 37°C in presence of 5% CO_2_ 1 h for gelation before adding organoid culture medium. They were then treated by BT. The growth of AM67 was monitored with EVOS M7000 Imaging System (Thermo Fisher Scientific) and quantified by ImageJ.

### 2.6 Transmission Electron Microscopy

Transmission electron microscopy was used for detection of organelle structure details within cells and was performed in the High‐Resolution Electron Microscopy Facility (HREMF) at MD Anderson. PANC‐1 cells (1 × 10^5^) were plated in 12‐well plates and then exposed to BT for 15 and 30 min, respectively. Immediately after BT, PANC‐1 cells were washed with PBS (without Ca^++^/Mg^++^) prior to treatment with EM fixative containing 3% glutaraldehyde and 2% paraformaldehyde in 0.1 M cacodylate buffer (pH 7.3) and then further processed according to the standard operating procedure of HREMF and procedures published previously [26]. Briefly, the cells were then washed in 0.1 M sodium cacodylate buffer and treated with 0.1% Millipore‐filtered cacodylate buffered tannic acid, postfixed with 1% buffered osmium and stained with 0.1% Millipore‐filtered uranyl acetate, prior to dehydration with increasing concentrations of ethanol followed by infiltration and embedding in LX‐112 medium. The samples were then polymerized in a 60°C oven for approximately 3 days. Ultrathin sections were cut using a Leica Ultracut microtome (Leica, Deerfield, IL) and stained with uranyl acetate and lead citrate in a Leica EM Stainer. The samples were examined in a JEM 1010 transmission electron microscope (JEOL USA Inc., Peabody, MA) using an accelerating voltage of 80 kV. Digital images were obtained using an AMT imaging system (Advanced Microscopy Techniques Corp, Danvers, MA). Quantification of mitochondrial width and length of 30 mitochondria in 6 cells (*n* = 5 per cell) under each treatment condition was determined using ImageJ software (NIH) as described by Lam et al. [27]. ImageJ software converts pixel counts to nm which helps to measure mitochondrial width and length. The Freehand tool and ROI manager from ImageJ were used to quantify the changes of mitochondria.

### 2.7 Cell Cycle Analysis

For cell cycle analysis, cells (2.5 × 10^5^) cultured in 6‐well plates were exposed to BT or SC for 15 min. Cells were immediately trypsinized and centrifuged. The cell pellets were suspended, washed with 1× PBS, and fixed in 70% ethanol at 4°C overnight. The fixed cells were further washed with 1× PBS and suspended in PBTB staining solution containing PBS, propidium iodide (5 μg/mL), and DNase‐free Rnase A (10 μg/mL). Cells were incubated in the dark at room temperature for 15 min and put on ice until data were acquired using a BD FACS Caliber flow cytometer (BD Biosciences, San Jose, CA). The percentage of cells in each phase of the cell cycle was determined from the DNA histogram content.

### 2.8 Cell Voltage Characterization

Briefly, cells (5–10 × 10^3^) were plated the day prior to treatment. After the treatment, plates were cultured for 1 h or 5 days in the incubator at 37°C in 5% CO_2_ and 95% humidified air. DiBAC4 [3], a slow‐response potential‐sensitive probe, was used to stain and measure the depolarization of the cells [28, 29, 30]. The working DiBAC4 [3] solution was prepared by mixing the stock solution (1 mg/ml DMSO) with Dulbecco modified Eagle medium–free fetal bovine serum at a ratio of 1:4000. Cells were washed with PBS and DiBAC4 [3] working solution together with 1 drop of NucBlue Live Ready Probes Reagent and incubated for 15 min at 37°C. Green fluorescent protein and DAPI images were captured by the EVOS M5000 Imaging System (AMF5000) or EVOS M7000 Imaging System (AMF7000). Fluorescence intensity was quantified by ImageJ for the images captured by EVOS M5000 or by Invitrogen Celleste software for the images captured by EVOS M7000.

### 2.9 Cell Migration and Invasion

Colorimetric cell invasion assay was performed using Cytoselect, the 24 wells basement membrane kit (Cell Biolabs, #CBA‐110) to visualize tumor cell invasion. Briefly, PANC‐1 cells suspension (1.0 × 10^6^, 300 μL) were plated in polycarbonate inserts/upper chamber with basement membrane (8 μm pore size) in DMEM without FBS whereas the bottom chamber contained 500 μL DMEM with FBS. Control cells and treated cells were plated in two different plates. After BT (15 min or 30 min), the cells were continually incubated for 48 h at 37°C in presence of 5% CO_2_. The media were aspirated carefully from the inserts after 48 h and the inserts were transferred to clean wells containing 400 μL of staining solution from the kit for 10 min followed by gently washing with clean water. They were then air dried and images of invading cells were taken by EVOS M7000 Imaging System (Thermo Fisher Scientific).

Quantitative cell invasion assays were done using Cytoselect cell invasion assay kits (#CBA‐111, Cell Biolabs, USA) in 24‐well plate format. 1.0 × 10^6^ cells in 300 μL medium without fetal bovine serum were plated in each insert (6 inserts/group). An aliquot of 500 μL of media containing 10% fetal bovine serum was used in the lower well of the invasion plate. The plates were then exposed to BT (15 min or 30 min) followed by incubation for an additional 48 h in a humidified CO_2_ incubator. The inserts were processed as described in the manufacturer's protocol. Similarly, the cell migration assay was conducted using the CytoSelect Cell migration Assay kit (24‐well format, CBA‐102, Cell Biolabs), which uses basement membrane‐coated inserts to assay the migration properties of tumor cells. The endpoint was monitored 24 h after BT. The fluorescence intensity was measured with a microplate reader at 480 nm/520 nm (Molecular Device, San Jose, CA).

For the scratch assay, the Culture‐Insert 2 Well in μ‐Dish 35 mm (Ibidi, Fitchburg, WI) was used. The dish has culture‐inserts to create a reproducible gap size. MiaPaca‐2 cells (6 × 10^5^) were seeded and cultured in each of the two culture‐insert wells for 24 h. The insert was then gently removed by sterile tweezers and an aliquot of 1 mL of culture medium was added to the culture dishes. The cell culture dishes were placed in two separate CytoSmart FL microscopes (Axion Biosystem, Atlanta, GA) inside two incubators, and the cell culture dishes were exposed to SC or BT. Images were captured every 30 min for 48 h. The images were analyzed by CytoSmart scratch assay online program.

### 2.10 Reverse Phase Protein Array (RPPA)

Immediately after being exposed to BT for 15 or 30 min, PANC‐1 cells were lysed in the lysis buffer provided by the Functional Proteomics Core Facility at MD Anderson. Cell lysates were then subjected to Reverse Phase Protein Array (RPPA) analysis by the Functional Proteomics Core Facility at MD Anderson as described previously [24].

### 2.11 Immunoblotting

Immunoblotting was performed using an automated Western blotting system: the Jess Simple Western system (ProteinSimple, San Jose, CA) automatically separates and immunodetects proteins by size through its capillaries. To quantify the protein expression of interest in the treated cells, we followed the standard method for the 12–230 kDa Jess separation module (SM‐W004). The final protein concentration of tissue lysate (0.4 to 1 mg/mL) was obtained by mixing measured cell lysate (0.6 to 2 mg/mL) with 0.1× sample buffer and Fluorescent 5× Master mix (ProteinSimple). This mixture was denatured at 95°C for 5 min. Primary antibodies of interest are indicated in the Results section, and β‐actin or tubulin were prepared by mixing antibody diluent at a ratio of 1:10 to 1:50. Secondary antibodies were prepared by combining chemiluminescence antibody and fluorescence antibody. After that, cell lysate samples were loaded into the Ladder plate (12–230 kDa), followed by loading of antibody diluent, primary antibody, secondary antibody, a luminol‐s and peroxide mixture (for chemiluminescence detection), and wash buffer according to the manufacturer instructions. Digital images of chemiluminescence and fluorescence for the proteins of interest were captured with Compass Simple Western software (version 4.1.0, ProteinSimple), and the area was used for quantification of the proteins.

### 2.12 FOXM1 Gene Modification

To establish FOXM1‐overexpressing PANC‐1 cells, we used hFOXM1 human ORF clone lentiviral particles purchased from OriGene USA (Rockville, MD) to overexpress the FOXM1 gene in PANC‐1 cells. Lentiparticles were thawed on ice and added to 6‐well plates directly in the medium (500 μL) along with Polybrene (final concentration 8 μg/mL), and the lentiparticles were then incubated for 20 h at 37°C in a humidified CO_2_ incubator. To generate stable cell lines, we plated transduced cells at low densities in a fresh 6‐well plate, and the clones were selected with puromycin (10 μg/mL). Single colonies were picked, expanded further, and characterized using Jess/Western blot.

To develop the FOXM1 knockdown stable PANC‐1 cell lines, we transfected three sets of human FOXM1 hCMV‐TurboRFP shRNA lenti‐viruses (Horizon Discovery Bioscience) into PANC‐1 cells. After transfection for 48 h, puromycin (10 μg/mL) was used for the colony selection for at least 2 weeks. The colony characterized with downregulated FOXM1 was then cultured with 10 ng/mL puromycin for all additional experiments. hFOXM1 knockdown was confirmed by Western blot (Jess analysis), and PANC‐1 cells that were most efficiently knocked down on FOXM1 were chosen for the experiments.

FOXM1 knockout L3.7 cells were generated by CRISPR‐Cas9 transfection. Briefly, L3.7 cells were plated in 6‐well plates and allowed to attach overnight. The transfection of a scramble control vector or a FOXM1 knockout gene kit via CRISPR‐Cas9 (OriGene, cat. #KN402246) was carried out using TurboFectin Transfection Reagent (OriGene, cat. #TF81001) following the manufacturer's instructions. Briefly, 1 μg of one of the gRNA vectors, gRNA1 or gRNA2 in 250 μL of Opti‐MEM I (Life Technologies), in separate tubes, was vortexed gently. Then 1 μg of the donor DNA was added into the same 250 μL of Opti‐MEM I. An aliquot of 6 μL of Turbofectin 8.0 was added to the diluted DNA and pipetted gently to mix completely. The mixture was incubated for 15 min at room temperature. The mixture above was added dropwise to the cells plated in 6‐well plates. The plate was gently rocked back and forth and side to side to distribute the complex evenly. Following that, cells were incubated in a 5% CO_2_ incubator. Then 48 h after transfection, cells were split at 1:10, grown for an additional 3 days, and split again at 1:10. This procedure was repeated seven times in total. After passage 7, puromycin was added and expression was checked using Jess. The colonies with stable FOXM1 knockout (g2‐1; Figure S1) were selected with puromycin and by substantially reduced expression of FOXM1.

To establish the inducible FOXM1 expressing PANC‐1 or COLO357 cells, the tetracycline inducible pCW57.1 HA‐ FOXM1b was purchased from Addgene (Watertown, MA). The inducible pCW57.1 HA‐ FOXM1b (5 mg) was transiently transfected to HEK293T cells in 10 cm plate, along with helper plasmids psPAX2 (6 mg) and 2 mg of pMD2G (Addgene: 12260, Addgene: 12259) using X‐tremeGENE9 DNA (Roche) transfection reagent as per the manufacturer's protocol. Viral supernatants were collected by filtering through 0.45 μm filter (Millipore) after 48 h of transfection. The PANC‐1 cells were transduced with the viral supernatant in presence of Polybrene (4 μg/mL, Sigma) in serial dilutions in 10 cm plates. The plates were then treated with 0.5 μg/mL Puromycin 48 h post transfection and puromycin selection was continued for 5 days. After 5 days, cells were cultured without Puromycin for a week. To test the doxycycline (Sigma) inducible human FOXM1 expression in PANC‐1 or COLO357 cells, the cells were plated in six well plates and treated with/without doxycycline (250, 500 and 1000 ng). Media with or without doxycycline were changed every 24 h. The expression of inducible FOXM1 was monitored using automated western blot system (Jess, Protein Simple) after 72 h of doxycycline treatment.

### 2.13 Animal Models of Pancreatic Cancer

#### 2.13.1 Animals

Animal studies were conducted according to MD Anderson Cancer Center Animal Care and Use Committee (IACUC) rules and regulations under approved IACUC animal care protocol (00001212‐RN03). Animals were housed in facilities approved by the Association for Assessment and Accreditation of Laboratory Animal Care International in accordance with the current regulations and standards of the US Department of Agriculture and Department of Health and Human Services.

For tumorigenesis of human PANC‐1 cells, we used pathogen‐free female athymic BALB/c nude mice (6 to 8 weeks old) with body weight 22 ± 5 g, purchased from the Jackson Laboratory. Each mouse received an intrapancreatic injection of PANC‐1 cells at 0.5 × 10^6^ cells per 50 μL of Hank balanced salt solution. Ten days after injection, all animals were imaged using a magnetic resonance scanner in the Small Animal Imaging Facility at MD Anderson Cancer Center per standard protocol for confirming pancreatic tumor formation in mice and then randomized to ensure balance of tumor size among groups before the start of treatment.

For the study with KPCY mouse pancreatic tumor model, KPCY 6419c5 cells (1 × 10^4^) was suspended with 50 μL of Matrigel and injected into the parenchyma of the pancreas. T2‐weighted MRI images were acquired on mice to monitor the size of pancreatic tumors 10–14 days after injection, and they were then randomized to ensure balance of tumor size among groups before the start of treatment.

#### 2.13.2 Procedures

Two separate PANC‐1 animal studies were conducted. Mice with tumor size 1–10 mm^2^ were randomized into four groups (*n* = 16 mice per group for study #1 and *n* = 30 mice per group for study #2): (1) BT treatment group, with treatment provided 30 min per day, three times per week, for 3 weeks; (2) SC group, which received the same treatment schedule as the BT group but a sham therapist mimicked the movements and distance from the animals used by the biofield therapist; (3) colony control (CC) group, in which the animals stayed in the animal facility the whole time; and (4) gemcitabine‐positive control group, which was treated with gemcitabine and also stayed in the animal facility the whole time. Both BT and SC groups of mice were taken from the animal colony to the laboratory in their home cages, where they underwent the treatment sessions on a bench maintained in their home cages (3–5 mice per cage). For the gemcitabine‐positive control group, gemcitabine was injected intraperitoneally at 40 mg/kg in saline three times per week and mice were maintained in the home colony. Similarly, mice from the CC group were injected with the same volume of saline intraperitoneally three times per week and maintained in the home colony.

The mice were treated for 3 weeks and then observed for another 7 weeks. Body weight of each mouse was measured once a week. At the termination of the study, mice were euthanized by exposing them to CO_2_, which is consistent with the recommendation of the Panel on Euthanasia of the AVMA and is an accepted practice approved by the Animal Care and Use Committee at The University of Texas MD Anderson Cancer Center. Blood, tumor/pancreas, spleen, liver, and lungs were collected and either flash‐frozen or fixed in 10% formalin for further analysis.

For the study with KPCY mouse pancreatic tumor model, randomization to the same four groups (*n* = 16 mice per group) was based on tumor size before the start of treatment to ensure balance among groups and the treatment schedule was similar to that of the PANC‐1 animal model. Mice were euthanized 24 h after the last treatment. Primary tumor and liver tissues were collected and weighed. The nodules in the liver tissues were examined macroscopically and the intensity of YFP+ image of the liver was determined by imaging with the Perkin Elmer IVIS Spectrum instrument (Caliper Lifesciences, Hopkinton, MA) in the Small Animal Imaging Facility of MDACC.

### 2.14 Immunohistochemical Analysis

Liver tissue samples from mice were obtained at necropsy. Tissues were fixed in 10% neutral buffered formalin and subsequently embedded in paraffin. Sections were stained with hematoxylin and eosin using a standard protocol of the Histopathological Core Facility of MD Anderson. The stained slides were scanned with Aperio AT2 bright‐field slide scanner and examined by a certified pathologist in a blinded fashion.

### 2.15 Statistical Analysis

GraphPad Prism was used for the statistical analysis (*t*–test or ANOVA) in both in vitro and in vivo studies. For the cell proliferation assay, *n* = 3 per treatment condition and the average of three individual plates was reported or *n* = 4 or 8 per treatment condition and the average of four wells was reported under the similar treatment condition. For the rest of the in vitro tests, *n* = 3–7 wells per group. For the comparison of liver tumor burden between SC and treatment group, we used Wilcoxon rank sum test due to non‐normal distributions. For the comparison of percentage of liver metastasis, Fisher's exact test was used. For RPPA data, statistical analysis was performed using R Statistical Software version 4.1.1 (R Foundation for Statistical Computing, Vienna, Austria) and Wilcoxon rank‐sum tests. For the in vivo studies, sample sizes randomized per group are noted above. The sample size for each study was determined based on our pilot data. Across all three studies, a few mice did not survive to the predefined study endpoints. Animals that died prematurely due to health‐related issues or experimental procedures were excluded from the final analysis. There were no significant differences between groups in mice that died prematurely. The final numbers analyzed per group are noted in Figure 8 as individual values or in the legend. For these analyses, *p* values < 0.05 were considered statistically significant. Data are presented as mean ± SD unless otherwise indicated. For all the analyses, the primary comparisons of interest were between the SC and BT groups. In these two conditions, all environmental factors are controlled, with the only difference between the groups being the administration of BT. The IC (in vitro) and CC (in vivo) groups were included as a quality control of the cells or animal models. Although not necessary to include these controls, this was an extra stringent control condition. However, differences between IC/CC and BT in the absence of SC would not be of interest or demonstrate the effects of BT per se as the conditions between IC/CC and BT were not the same.

## 3 Results

### 3.1 BT Decreased Cell Growth of Human and Mouse Pancreatic Cancer Cells and Patient‐Derived Organoids (PDOs)

**FIGURE 1:**
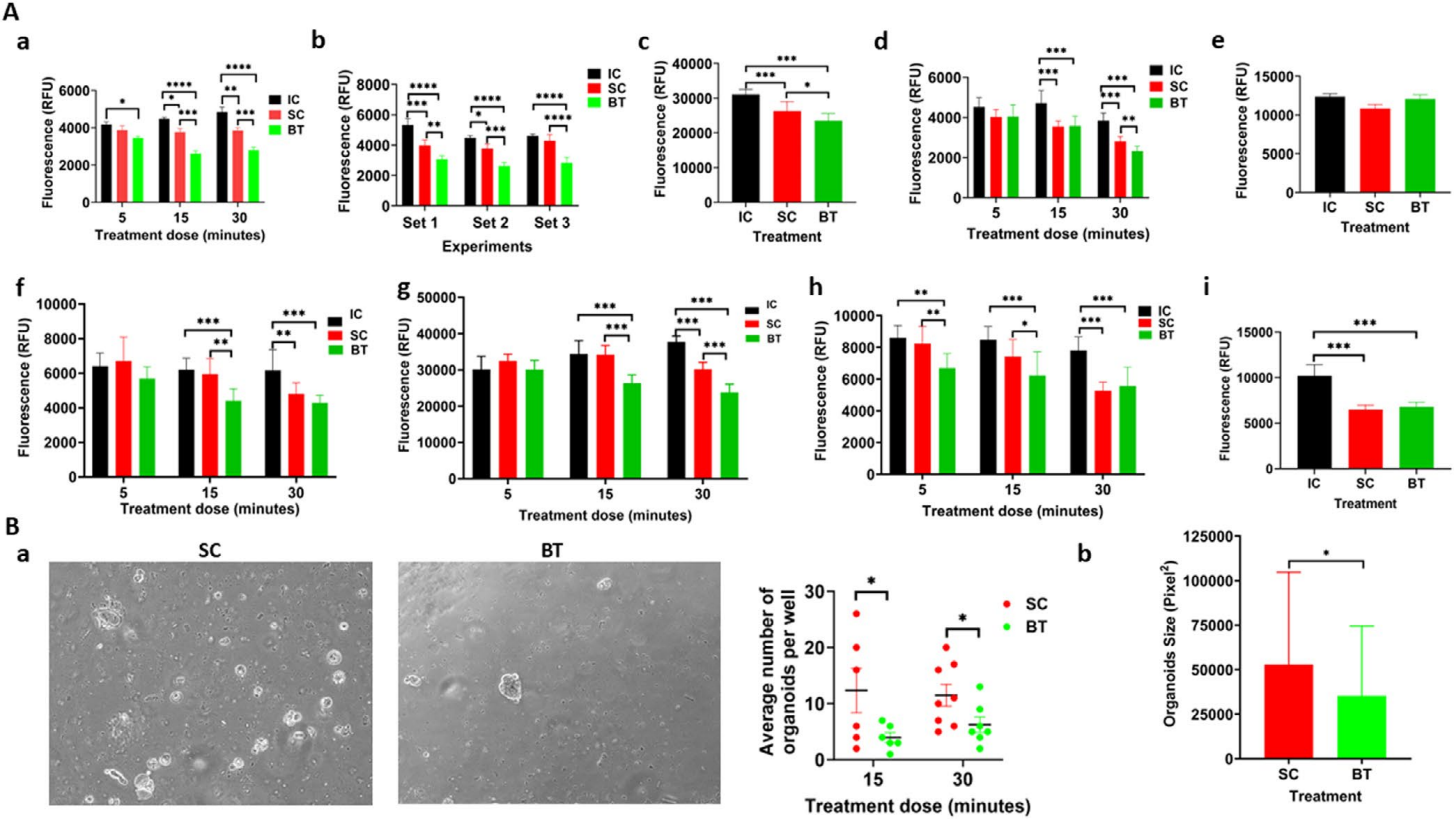
(A) Effect of biofield therapy (BT) on the growth of pancreatic ductal adenocarcinoma cells, including human PANC‐1 (a and b) and COLO357 cells (c), mouse Panc02 cells (d), and immortalized human pancreatic ductal epithelial cells (HPDE‐1/E6E7) (e) treated by biofield Therapist 1; PANC‐1 (f), COLO357 (g), L3.7 (h), and HPDE‐1/E6E7 cells (i) treated by biofield Therapist 2, compared with sham control (SC) and incubator control (IC). Data are presented as mean ± SE. (B) Effect of BT on the growth of human patient‐derived pancreatic cancer organoids (PDOs, AM67): (a) The average number of PDOs AM67 11 days after BT (15 and 30 min) or SC; (b) The size of the PDOs AM67 after BT treatment (15 min) or SC. Data are presented as mean ± SD (*p < 0.05; **p < 0.01; ***p < 0.001; ****p < 0.0001; *n* > 4).

We first examined the proliferation of various human and mouse pancreatic cancer cells after 5, 15, or 30 min of BT treatment with Therapist 1. As illustrated in Figure 1Aa, PANC‐1 cells exposed to BT for 15 and 30 min had moderately but significantly reduced cell growth at 1 h after BT compared with the IC and SC groups. Cell growth was also reduced in the SC group (i.e., the cells placed on the counter), but the reduction was greater for BT compared with both other groups. We repeated the experiments again using the 15‐min dose in more than 11 different experiments (three replicates shown in Figure 1Ab; five replicates shown in Figure S2A–C; all experiments conducted on separate days). Similar activity was also observed in human pancreatic cancer COLO357 (Figure 1Ac) and MiaPaCa‐2 cells (Figure S3A) after exposure to BT. In contrast to PANC‐1 cells, BT at the 30‐min dose seemed to show the greatest decrease in cell growth relative to that of the IC and SC groups in Panc02 cells (Figure 1Ad). We then treated the Panc02 cells with BT for 30 min in three separate experiments and BT consistently and significantly inhibited the growth of Panc02 cells compared with the IC and SC groups (Figure S2D–F). BT also significantly reduced the growth of KPCY (mouse pancreatic cancer) cells after 15‐min exposure (Figure S3B). To further understand whether the suppressive effect of BT on cell growth was specific to cancer cells, we exposed immortalized human pancreatic ductal epithelial cells (HPDE‐1/E6E7) to BT for 15 min and found HPDE‐1/E6E7 cells exposed to BT had a similar growth rate to that of IC group, but slightly higher than that of the SC group (Figure 1Ae).

PANC‐1, COLO357, and L3.7 cells were also treated by a second biofield therapist (Therapist 2, 15 min) and exhibited significantly slower growth relative to both IC and SC (Figure 1Af–h). The growth rate of HPDE‐1/E6E7 cells treated by Therapist 2 was similar to that of the SC group (Figure 1Ai), consistent with the results observed with Therapist 1. The PANC‐1 cells studies were further replicated with a third biofield therapist (Therapist 3) (Figure S4).

Given that PDOs represent the genetic and epigenetic landscape of each PDAC patient and are a reliable model to understand cancer biology and predict treatment, we further tested the growth of AM67 PDOs after BT. The number of PDOs in the BT group was significantly less than that of the SC group 11 days after BT (Figure 1Ba). Similarly, BT also led to a reduction in the size of the AM67 PDOs compared to that of the SC group (Figure 1Bb).

### 3.2 BT Altered the Ultrastructure of PANC‐1 Cells

**FIGURE 2:**
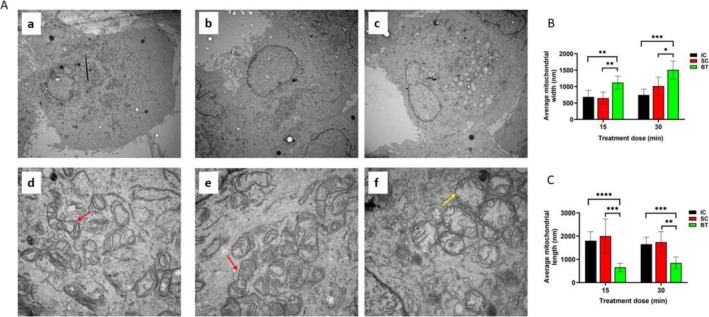
Ultrastructural changes of PANC‐1 cells after BT treatment measured by Transmission Electron Microscope (TEM). Transmission electron micrographs (A) showed morphologic changes in PANC‐1 cells in the incubator control (IC) group (a and d), sham control (SC) group (b and e), and biofield therapy (BT) group (c and f) after 15 min of exposure to treatment (samples were collected immediately after treatment). Note the normal appearance mitochondria (red arrows) in the IC and SC groups (d and e) and swollen mitochondria in the BT group (yellow arrow, f). Images a–c were taken at 5000×; images d–f were taken at 25,000×. Quantitative analysis of mitochondrial width (B) and length (C) in PANC‐1 cells (30 mitochondria in 6 cells (*n* = 5 per cell) under each treatment condition). Data are presented as mean ± SD (*p < 0.05; **p < 0.01; ***p < 0.001; **** *p* < 0.0001).

The ultrastructural changes in cellular organelles were examined immediately after BT treatment of PANC‐1 cells using Transmission Electron Microscope (TEM). As shown in Figure 2A, PANC‐1 cells in the IC (Figure 2Aa,d) and SC (Figure 2Ab,e) groups had elongated mitochondria with well‐organized cristae. In contrast, mitochondrial cristae were disordered, and mitochondria were markedly swollen in PANC‐1 cells exposed to BT (Figure 2Ac,f). Quantitative analysis of mitochondrial width and length showed that BT significantly increased the width (Figure 2B) and reduced the length (Figure 2C) of mitochondria compared to that of IC and SC, with no differences between IC and SC, further supporting that BT treatment led to mitochondria swelling and alteration of mitochondrial morphology. Similar mitochondrial changes, that is, losing the cristae integrity and noticeable swelling of the mitochondria, were observed after 15 (Figure S1) and 30‐min of BT treatment in repeated studies using PANC‐1 cells (Figure S5B). This profound and visually obvious morphology changes in the mitochondrial ultrastructure not only substantiates but also provides new insight into the biological action of BT on important cellular function.

### 3.3 BT Blocked the Cell Cycle in the G0/G1 Phases in Human PDAC Cells

**FIGURE 3:**
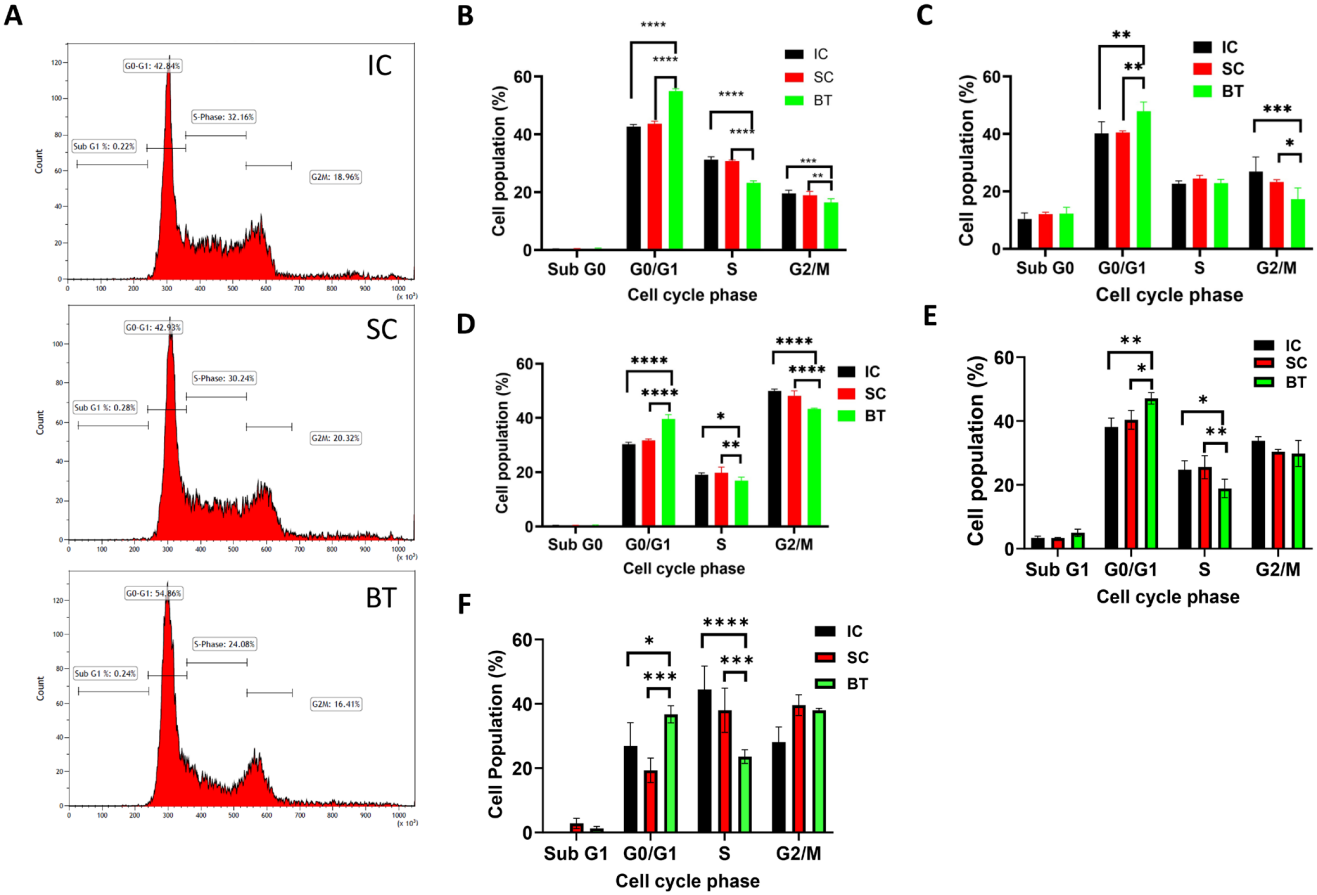
Cell cycle analysis of PDAC cells. (A‐C) PANC‐1 cells were plated in 6‐well plates overnight and then exposed to biofield therapy (BT), sham control (SC), or incubator control (IC) for 15 min. Cells were collected immediately after the treatment. Cell cycle analysis was carried out with propidium iodide staining and flow cytometry. (A) Histogram of cell cycle populations of PANC‐1 cells in the IC, SC, and BT groups after 15‐min exposures. (B, C) Quantitative analysis of PANC‐1 cells in different cell cycle phases after 15‐min BT, SC, or IC in two separate experiments. (D) PANC‐1 cells were plated in 6‐well plates for 48 h prior to BT (15 min). (E, F) L3.7 cells and COLO357 cells were plated overnight and then exposed to BT for 30 min. Data are presented as mean ± SD (*p < 0.05; **p < 0.01; ***p < 0.001; ****p < 0.0001; *n* = 3).

To further understand the biological changes of pancreatic cancer cells after BT, we performed cell cycle analysis of human PDAC cells. As shown in Figure 3, BT led to significant 1.5‐fold increases in G0/G1 phase cells in PANC‐1 cells compared with the IC and SC groups (Figure 3A,B). Similar studies were repeated three more times and the same changes in the cell cycle pattern were observed in all experiments (Figure 3C; Figure S6A,B). To confirm that the cell cycle changes observed in PANC‐1 cells after BT were independent of baseline cell growth, PANC‐1 cells were plated 2 days prior to treatment and were collected immediately after treatment. As shown in Figure 3D, even though the G0/G1 phase population of cells was lower and the G2/M phase population of cells almost doubled in the IC and SC groups compared with those shown in Figure 3B,C, PANC‐1 cells exposed to BT were again arrested in the G0/G1 cell cycle phase. BT also led to significant G0/G1 phase arrest in other human PDAC L3.7 (Figure 3E) and COLO357 cells (Figure 3F). Furthermore, PANC‐1 cells treated by Therapists 2 and 3 for 15 min also displayed significantly higher G0/G1 phase cell populations than those of the SC or IC groups (Figure S6C,D).

### 3.4 BT Reduced Pancreatic Cancer Cell Membrane Voltage Potential, Leading to Hyperpolarization

**FIGURE 4:**
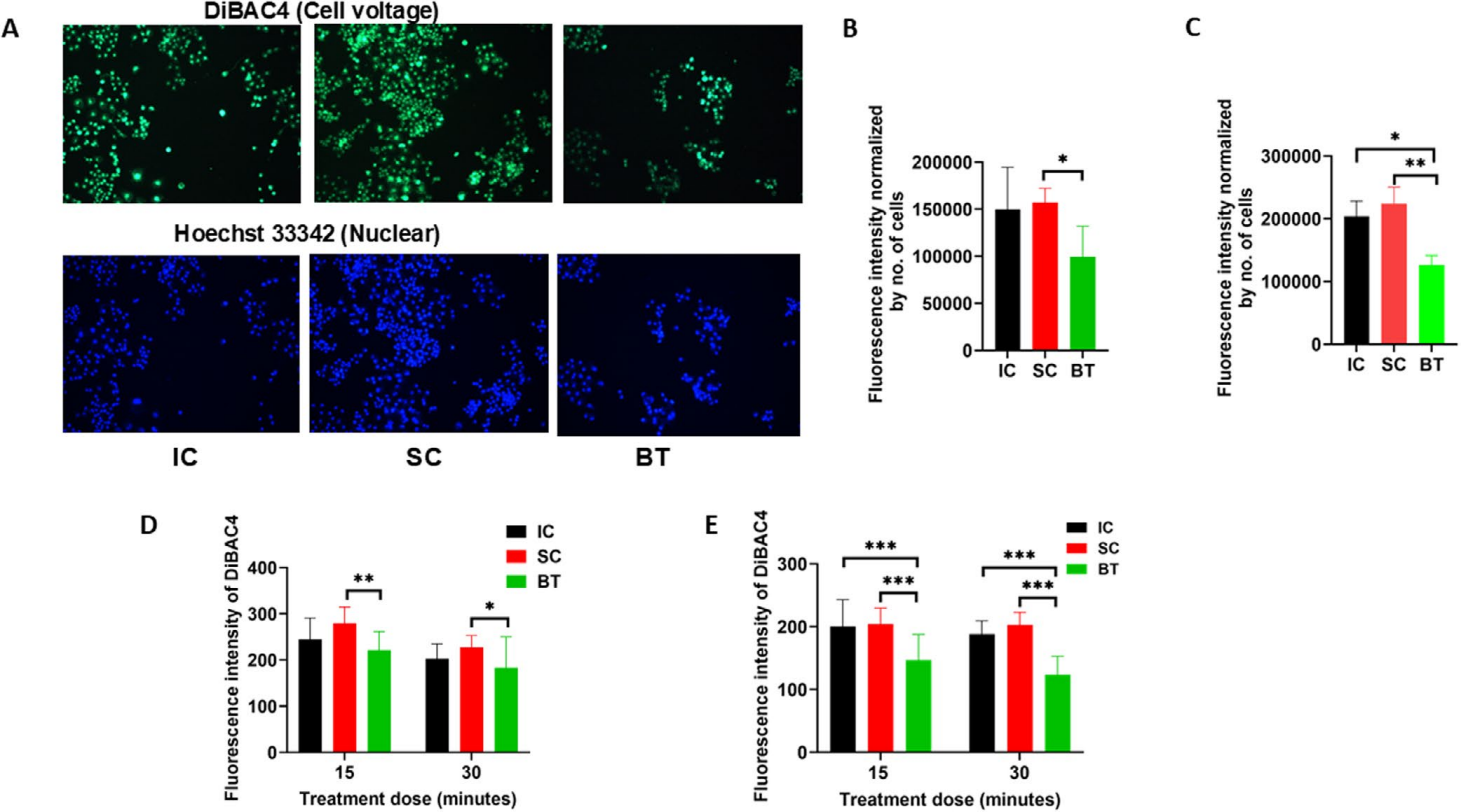
Cell membrane voltage potential of pancreatic ductal adenocarcinoma cells. (A) Representative fluorescence imaging of DiBAC4 and Hoechst in PANC‐1 cells under incubator control (IC), sham control (SC), and biofield therapy (BT, 15 min) conditions. (B) Quantitative analysis of cell voltage potential in PANC‐1 cells after 15‐min BT. (C) Quantitative analysis of cell voltage potential in PANC‐1 cells after 15‐min BT for two consecutive days. (D) Cell voltage potential of MiaPaCa‐2 cells measured at 1 h after BT. (E) Cell voltage of KPCY cells measured at 1 h after BT. Data are presented as mean ± SD (*p < 0.05; **p < 0.01; ***p < 0.001; *n* > 4).

Given the known roles of *V*_*m*_ in maintaining and suppressing the cancer phenotype [31, 32], we examined whether resting *V*_*m*_ could be part of the mechanism by which BT exerts its effects. As shown in Figures 4A and 4B, five days after exposure to BT, SC, or IC, IC and SC PANC‐1 cells had similar fluorescence intensity of DiBAC4, whereas PANC‐1 cells exposed to BT had significantly weaker staining relative to the other two groups (Figure 4A,B). When PANC‐1 cells were exposed to BT for two consecutive days, the *V*_*m*_ was reduced by almost 50% compared with that of the other two groups (*p* < 0.05 and *p* < 0.01, respectively; Figure 4C). The effects of BT on decreasing *V*_*m*_ were also detected in MiaPaCa‐2 cells (Figure 4D, Figure S7A) and KPCY cells (Figure 4E, Figure S7B) immediately or 1 h after BT (15 or 30 min). Together, these findings suggest that BT can alter *V*_*m*_, which may lead to hyperpolarization of pancreatic cancer cells.

**FIGURE 5:**
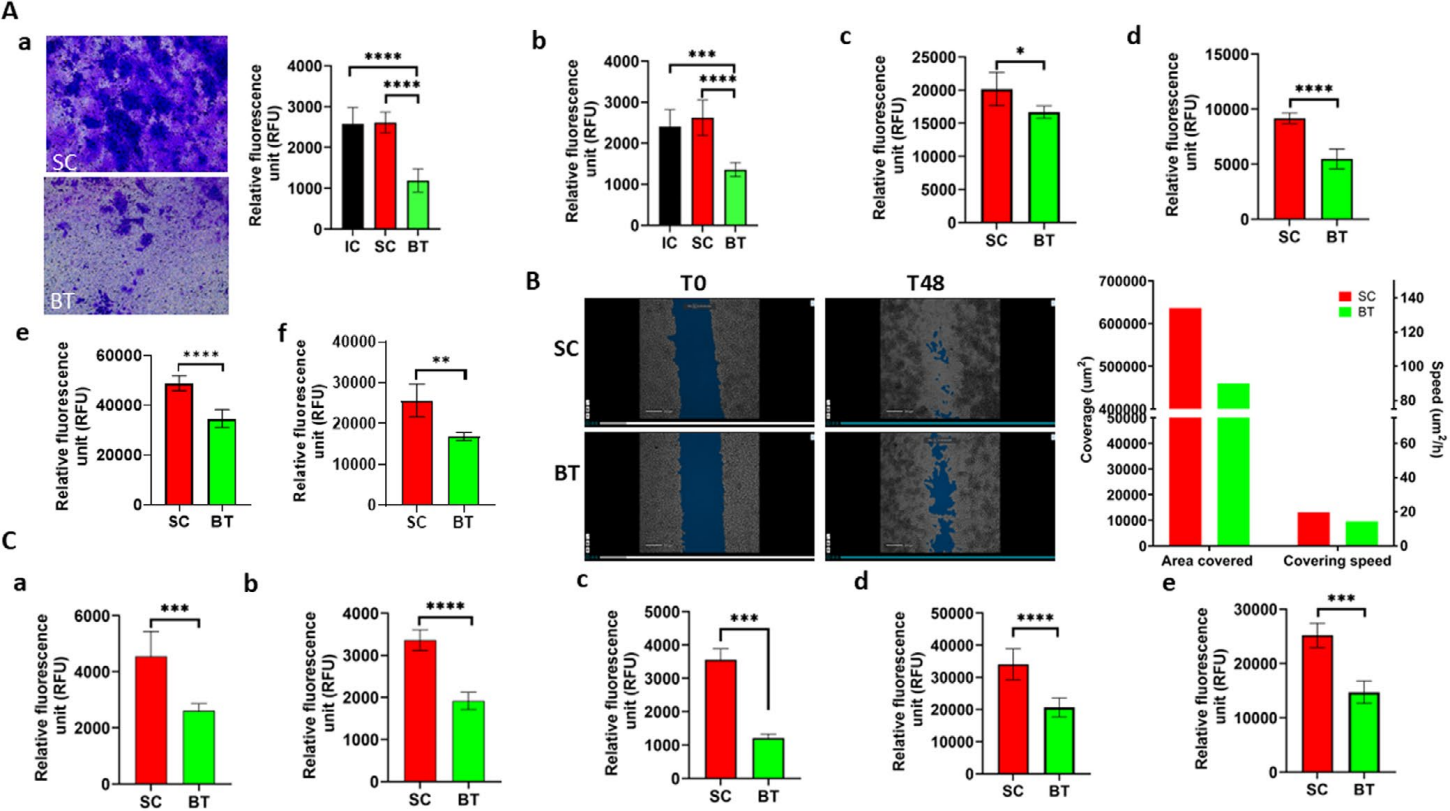
Biofield therapy (BT) markedly inhibited invasiveness and migration of PDAC cells compared with sham control (SC) and incubator control (IC). (A) Invasiveness of PDAC cells: PANC‐1 (a), L3.7 (b), COLO357 (c), and KPCY (d), and migration of PANC‐1 (e) and L3.7 (f) cells. (B) Migration of the MiaPaCa‐2 cells via scratch assay. (C) Invasiveness of PANC‐1 (a), COLO357 (b), and L3.7 (c) cells and migration of PANC‐1 (d) and L3.7 (e) cells treated by Biofield Therapist 2 (*n* = 5–6). Data are presented as mean ± SD (*p < 0.05; **p < 0.01; ***p < 0.001; ****p < 0.0001).

### 3.5 BT Inhibited Migration and Invasion in PDAC Cells

The effect of BT on the invasiveness of PDAC cells was also examined. As shown in Figure 5Aa, PANC‐1 cells exposed to BT displayed 55% less invasiveness compared with IC and SC cells (*p* < 0.0001), with no differences between the IC and SC groups. Given that there were no differences between IC and SC groups, we repeated the experiment four additional times on different days with the BT and SC groups, and the results consistently showed that BT led to significant suppression of the invasiveness of PANC‐1 cells (Figure S8A–D). Additionally, L3.7 cells exposed to BT exhibited a similar reduction in invasiveness to that observed in PANC‐1 cells (Figure 5Ab and Figure S7E). In contrast, COLO357 cells exposed to BT displayed less invasiveness (22%) than that seen in PANC‐1 cells (Figure 5Ac). BT also reduced the invasiveness of KPCY cells to a similar degree to that observed in PANC‐1 cells (Figure 5Ad).

Consistent with the results of the cell invasion assays, BT also led to a significant reduction of migration in PANC‐1 and L3.7 cells, by 34% and 41%, respectively, compared with SC (*p* < 0.001; Figure 5Ae,f). The inhibitory effect of BT on migration in MiaPaca‐2 cells was observed in the scratch migration assay (Figure 5B). The anti‐invasiveness and anti‐migrative effects of BT in these PDAC cells were tested with Therapist 2, and the results were similar to those observed with Therapist 1 (Figure 5C), indicating the replicability of these findings across studies and therapists.

To confirm that the BT‐elicited anti‐invasiveness in the PANC‐1 cells was not caused by the difference in incubators used for incubating SC and BT cells after the treatment, we examined the invasiveness of PANC‐1 cells without treatment when they were incubated in the SC and BT incubators. The invasive capacity of PANC‐1 cells in both incubators was similar (Figure S7F).

### 3.6 BT Modified Key Cell Signaling Proteins, Especially FOXM1

Reverse Phase Proteomic Array (RPPA) measurement in PANC‐1 cells was conducted to further understand the potential mechanisms involved in BT‐elicited effects compared with the changes observed in the SC group. This was done in a blinded fashion, with Core Facility members unaware of the experimental design or groups.

**FIGURE 6:**
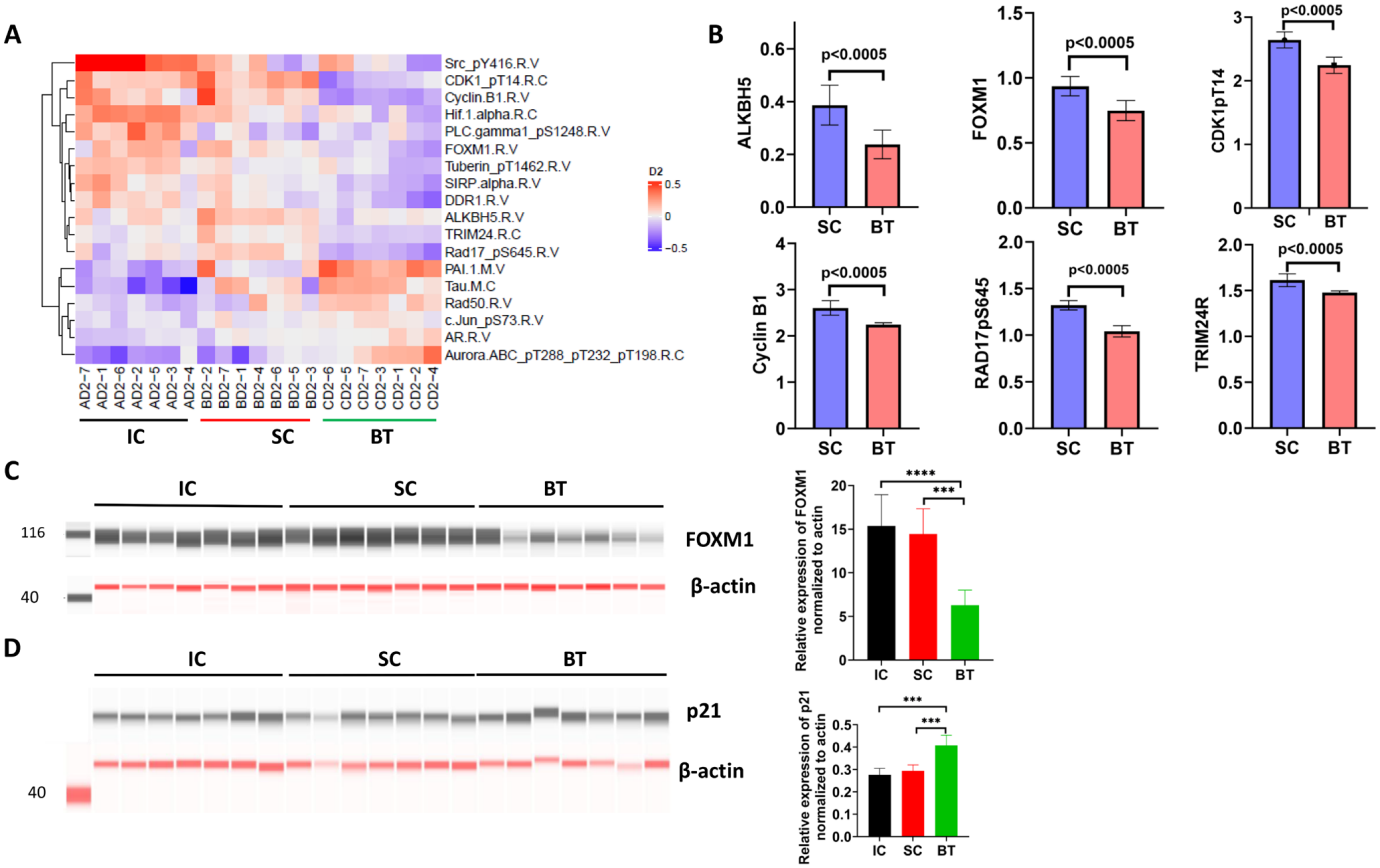
Expression of proteins that regulate the cell cycle and cell signaling in PANC‐1 cells exposed to biofield therapy (BT) for 15 min, compared with sham control (SC) and incubator control (IC). (A) Heatmap of cell cycle regulating proteins and cell signaling proteins in PANC‐1 cells, analyzed by reverse phase protein array (RPPA). The proteins included in this heatmap were statistically significant among different treatment groups according to Wilcoxon rank‐sum analysis, with *p* values lower than the Benjamini‐Hochberg thresholds for false discovery rate (< 0.05). (B) Expression of cell cycle regulating proteins and epigenetic modification proteins, such as CDK1, cyclin B1, FOXM1, and ALKBH5 in PANC‐1 cells exposed to BT compared with SC, as examined by RPPA (*n* = 7). (C‐D) Western blot analysis of FOXM1 (C) and p21 (D) in PANC‐1 cells exposed to BT for 15 min. Data are presented as mean ± SD (***p < 0.001; ****p < 0.0001; *n* = 7).

Although some proteins were upregulated by BT (Figure 6A), 15‐min BT significantly downregulated ALKBH5 (a known m6 demethylase and epitranscriptional regulator for FOXM1) by 41%. FOXM1 was downregulated by 21%, and cyclin B1 and pCDK1 were each downregulated by 15% (*p* < 0.0005 for all proteins; Figure 6B). Similar reductions in the expression of cyclin B1 and FOXM1 were observed after 30‐min BT in PANC‐1 cells, along with reductions in TRIM24, TFRC, and PAI‐1 M (Figure S9). The significant downregulation of FOXM1 protein by BT (15‐min) in PANC‐1 cells was further confirmed by automated Western Blot analysis (Jess method; Figure 6C). Because p21 arrested the cells in both the G1 and G2/M phases, we examined whether BT also affects p21 proteins. As shown in Figure 6D, BT indeed upregulated p21 expression in PANC‐1 cells. Similar results were observed in L3.7 cells exposed to BT (Figure S10). These findings suggest that BT can potentially affect the cell cycle of PANC‐1 cells by modulating the expression of key cell cycle regulators such as FOXM1 and p21.

### 3.7 BT Altered Epithelial‐Mesenchymal Transition (EMT) Related Markers in PANC‐1 Cells

To understand how BT suppressed the invasiveness of the PDAC cells, we examined EMT related marker expression in PANC‐1 cells. As shown in Figure S10, the expression of E‐cadherin, a marker of epithelial cells, in PANC‐1 cells exposed to BT was significantly higher than in the IC and SC groups (Figure S11A). In contrast, N‐cadherin, a marker of mesenchymal cells, was significantly lower in the BT group than in the IC and SC groups (*p* < 0.05; Figure S11B), suggesting that BT influenced PANC‐1 cell EMT, a process important for cancer cell invasion and metastasis.

### 3.8 Modulation of FOXM1 Expression Altered the Response of PDAC Cells to BT

**FIGURE 7:**
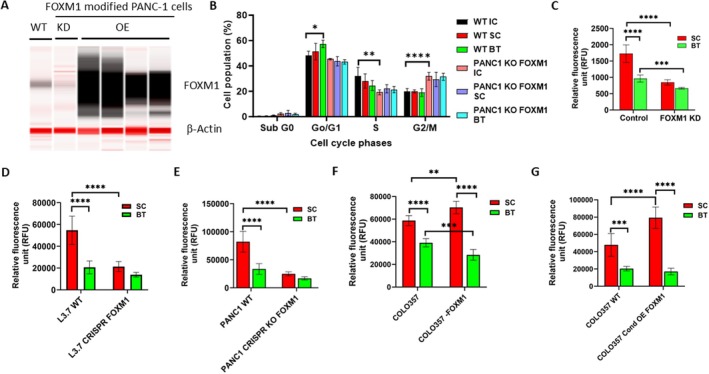
Modification of FOXM1 in biofield therapy (BT) elicited cell cycle alteration and anti‐invasiveness in PDAC cells. (A) Western blot analysis of FOXM1 in PANC‐1 cells (WT), FOXM1 stable‐knockdown PANC‐1 cells (KD), or FOXM1‐overexpressing (OE) PANC‐1 cells. (B) Cell cycle of FOXM1 knockout (KO) PANC‐1 cells exposed to BT compared with parental PANC‐1 cells. (C) Invasiveness of parental PANC‐1 cells and FOXM1‐knockdown (KD) PANC‐1 cells exposed to BT. (D) Invasiveness of wild‐type (WT) L3.7 cells and FOXM1‐knockout (KO) L3.7 cells exposed to BT. (E) Invasiveness of WT PANC‐1 cells and FOXM1‐KO PANC‐1 cells exposed to BT. (F) Invasiveness of control COLO357 cells and FOXM1 stably overexpressing COLO357 cells exposed to BT. (G) Invasiveness of control COLO357 and Tet‐inducible FOXM1 overexpressing COLO357 cells exposed to BT. Data are presented as mean ± SD (*p < 0.05; **p < 0.01; ***p < 0.001; ****p < 0.0001).

To determine whether FOXM1 is a potential molecular target responsible for BT‐elicited changes, we first examined the correlation between the ability of BT to downregulate FOXM1 protein expression and the suppressive effect of BT on the invasion of PDAC cells. As shown in Figure S12, the correlation was high (*R* ^2^ = 0.85) suggesting that the stronger the suppressive effect of BT on the expression of FOXM1 the greater the inhibition of BT on cell invasion. BT had the greatest effect in suppressing FOXM1 in PANC‐1 cells (> 40%) with the highest inhibition of invasion (54%) and BT did not suppress FOXM1 in COLO357 cells which had the least inhibition of the cell invasion (17.3%). This suggests that BT‐induced anti‐invasiveness in PDAC cells might be at least in part mediated by its ability to downregulate FOXM1 protein. We then examined the cell cycle and invasiveness of control PDAC cells and FOXM1‐modified PDAC cells after BT or SC. In the FOXM1 stable knockdown (KD) PANC‐1 cells, the expression of FOXM1 was reduced by almost 90% compared with that of parental PANC‐1 cells (Figure 7A). The BT treated parental PANC‐1 cells showed a significantly higher population of G0/G1‐phase cells compared with IC or SC, whereas no difference on G0/G1 phase cells was observed in BT treated CRISPR FOXM1 KO PANC‐1 cells compared to that of the IC or SC group (Figure 7B).

When the parental PANC‐1 cells were exposed to BT (15 min), the invasiveness of these cells was significantly reduced relative to SC (*p* < 0.001; Figure 7C), and relative to previous experiments (Figure 5Aa, Figure S7A–D). FOXM1 knockdown reduced the invasiveness of the PANC‐1 cells by almost 50%, which is similar to the effects of BT, and there were no differences in invasiveness between the BT and SC groups (Figure 7C). Although BT led to a significant 62.2% reduction of invasiveness in parental L3.7 cells relative to SC (Figures 7D and 5Ab, Figure S8E), CRISPR FOXM1 KO L3.7 cells significantly reduced invasiveness by 61% compared with parental L3.7 cells, and there were no longer differences between the BT and SC groups (Figure 7D). Similarly, FOXM1 KO PANC‐1 cells displayed a significantly lower number of invaded cells compared to that of parental PANC‐1 cells, and again BT‐induced suppression of PANC‐1 cell invasiveness was minimized after knocking out FOXM1 (Figure 7E), which was consistent with the FOXM1 KO L3.7 cell outcome (Figure 7D).

Conversely, overexpressing FOXM1 in COLO357 cells significantly increased the number of cells invaded by 19% compared with that of parental COLO357 cells. Additionally, BT led to a significant 33.6% reduction of cell invasiveness in parental COLO357 cells, whereas FOXM1‐overexpressing COLO357 cells showed significantly reduced invasiveness, by about 59.5% compared with the SC group (Figure 7F). We also investigated the anti‐invasiveness of BT in PANC‐1 and COLO357 cells with conditional over‐expression of the FOXM1 gene using the Tet‐on tetracycline (or doxycycline) inducible system. Again, conditional overexpressing FOXM1 in COLO357 cells significantly increased the number of cells invaded by 65.6% compared with that of parental COLO357 cells. BT led to a significant 57.4% reduction of cell invasiveness in parental COLO357 cells, whereas FOXM1‐overexpressing COLO357 cells showed significantly reduced invasiveness by about 78.4% compared with the SC group (*p* < 0.0001) (Figure 7G). Similar changes were also observed in FOXM1 stably overexpressing and conditional overexpressing PANC‐1 cells after being treated with BT (Figure S13A,B). These data suggest that FOXM1 might be at least partially responsible for the activity of BT as it relates to changes in the cell cycle and cell invasiveness in PDAC cells.

### 3.9 BT Inhibited Liver Metastasis of PANC‐1 and KPCY Cells in an Orthotopic Mouse Model

**FIGURE 8:**
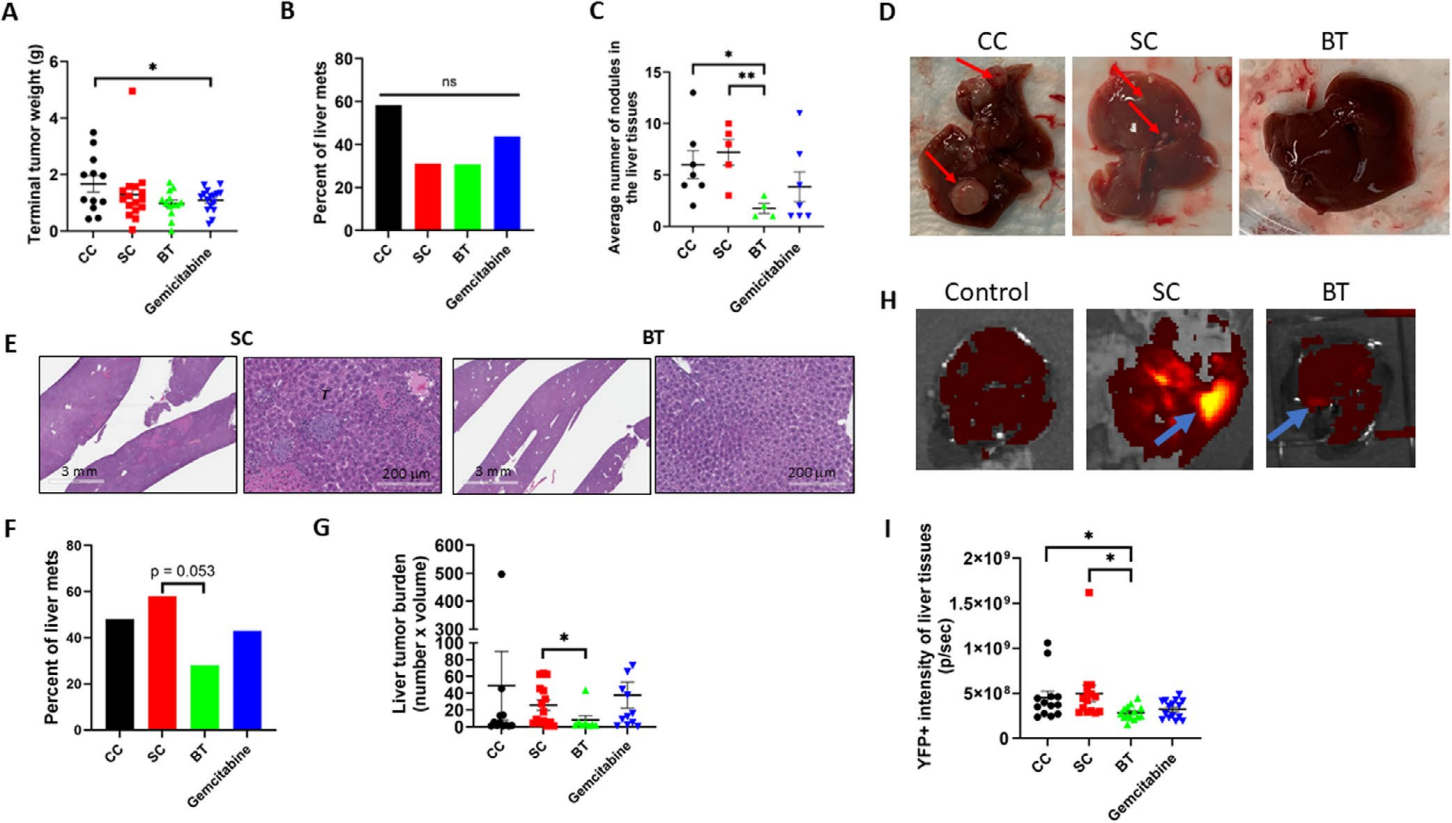
Biofield therapy (BT) inhibited the growth of primary pancreatic tumors and significantly reduced liver metastasis compared with sham control (SC) and colony control (CC) in a human PANC‐1 cell and mouse KPCY cell mouse orthotopic model. (A) Terminal tumor weight of PANC‐1 tumors after exposure to BT, control, or gemcitabine for 3 weeks and observation for an additional 7 weeks (Study #1). (B) Percentage of the PANC‐1 tumor‐bearing mice with liver metastasis at the termination of the study (*n*'s: CC = 12; SC = 16; BT = 13; gemcitabine = 16). (C) Average number of liver nodules in PANC‐1 tumor‐bearing mice with liver metastasis (Study #1). (D) Representative pictures of liver tissues collected from CC, SC, and BT mice. Red arrows indicate liver nodules. (E) Representative hematoxylin and eosin–stained images of mouse liver tissues in the SC and BT mice bearing PANC‐1 orthotopic tumors (Study #1). (F) Percentage of the PANC‐1 tumor‐bearing mice with liver metastasis at the termination of the study (Study #2; *n*'s: CC = 27; SC = 26; BT = 28; gemcitabine = 28). (G) Average liver tumor burden in PANC‐1 tumor‐bearing mice with liver metastasis in Study #2. (H) Representative liver YFP+ fluorescence images of normal control mice and mice bearing KPCY 6419c5 mouse tumor from SC and BT groups (Study #3). Control liver tissue was from the C57B/L6 mice without injection of tumor cells. Blue arrows indicate liver nodules with increased YFP imaging signal. (I) Liver metastasis in KPCY 6419c5 mouse model quantified by imaging the YFP fluorescence intensity in the liver tissues (Study #3). Data are presented as mean ± SE (*p < 0.05; **p < 0.01).

To further evaluate the antitumor effect of BT on pancreatic cancer, we conducted an antitumor efficacy study in a human PANC‐1 mouse orthotopic model. As shown in Figure 8A, the average terminal tumor weight of mice exposed to BT was 41% and 24% less than that of the CC and SC groups, respectively. However, the difference did not reach statistical significance. Gemcitabine (one‐quarter maximum tolerated dose, 40 mg/kg) led to a significant 35% reduction of the growth of PANC‐1 orthotopic tumors relative to the CC group (*p* < 0.05), with no differences between the gemcitabine and BT groups (Figure 8A). We also observed that 30% of the mice in the BT group had liver metastasis, which was 47% less than in the CC group (58.3% had liver metastasis), but no differences between SC and BT groups (Figure 8B). When the number of liver nodules in the mice with liver metastasis was examined macroscopically, the average number of liver nodules of mice exposed to BT was 1.8 ± 0.9 per liver, which was 70% and 75% less than that of the CC group (6.0 ± 3.6) and SC group (7.2 ± 2.8; *p* < 0.05), respectively. Mice treated with gemcitabine also had 36% fewer liver nodules compared with the CC group, but the difference did not reach statistical significance (Figure 8C). Although there were more and larger liver nodules in the CC mice and SC mice, a normal‐looking liver was present in the BT mice (Figure 8D).

Histopathologic examination (Figure 8E) of the liver nodules showed that the average number of liver nodules in the CC group was 16.3, compared with 8.7 in the BT group, which was also almost 50% less than in the SC group. When the study was repeated with the same therapist in a similar PANC‐1 mouse orthotopic model, 28% of BT mice had visible liver nodules, whereas liver metastasis was observed in 44% of CC mice, 58% of SC mice, and 43% of gemcitabine mice (Figure 8F; SC vs. BT, *p* = 0.053). Furthermore, BT significantly reduced liver tumor burden by 69% compared with SC (*p* < 0.05; Figure 8G), which was similar to our observations in the first study.

Similarly, when mice bearing KPCY (6419c5) tumors were treated with BT, the image of liver tissues showed higher YFP+ intensity in the SC mice compared to that of the BT treated mice (Figure 8H). The fluorescence intensity of BT treated liver tissues was statistically significantly lower than that of the CC and SC groups (*p* < 0.05), with no differences between BT and gemcitabine groups (Figure 8I). As there was one mouse in the SC group that was likely an outlier (more than 3 standard deviations from the mean; see Figure 8I), we took an extra conservative approach and reran the analyses without this subject. The difference between the SC and BT groups remained statistically significant.

## 4 Discussion

This series of studies provides several lines of evidence that BT may exert antitumor‐related effects in PDAC cells as well as in orthotopic mouse models using PANC‐1 and KPCY cells. BT consistently inhibited the proliferation and invasiveness of various human and mouse PDAC cells and PDOs in vitro, which was accompanied by pronounced alteration in mitochondrial morphology, significant induction of cell cycle arrest, reduction of the cell membrane voltage potential, and changes in epithelial‐mesenchymal transition marker expression. These findings were replicated across multiple experiments using stringent experimental controls, including incubator/colony controls and SCs, and involved three biofield therapists. BT moderately inhibited primary tumor growth, but significantly reduced liver metastasis in the PANC‐1 orthotopic mouse model in two separate studies and in the KPCY orthotopic mouse model, suggesting that BT may slow the growth of PANC‐1 and KPCY tumors and, more importantly, may influence metastatic spread to the liver. The findings from the animal models were consistent with the in vitro findings, both indicating that the magnitude of the effect of BT was stronger in terms of the anti‐invasiveness (see Figure 6) than for slowing the growth of PDAC cells (see Figure 1). Additionally, our findings showed that BT significantly reduced FOXM1 and its signaling loop–related protein expression in PANC‐1 cells. Upregulation, knockdown, and knockout of FOXM1 expression significantly modified the impact of BT on the cell cycle and invasiveness of PDAC cells. Taken together, these in vitro and in vivo data showed for the first time that BT may modify the growth and invasiveness of PDAC cells, at least in part, through downregulation of FOXM1, an important regulator of the cell cycle, metastasis, and stemness of pancreatic cancer. These findings indicate that BT warrants further exploration as a potential approach in pancreatic cancer.

PDAC is known to harbor a plethora of genetic and epigenetic alterations, including activation of a *KRas* oncogene mutation and inactivation of *CDKN2A*, *TP53*, and *SMAD4* tumor suppressors [33, 34]. Thus far, targeting these alterations for therapeutic intervention has not yielded significant clinical benefit, often due to pathway detour or the development of resistance. Alternatively, researchers are identifying and targeting downstream key effectors of these oncogenic signaling pathways. The FOXM1 transcription factor has been identified as such an effector because multiple key oncogenic pathway signals converge to activate the expression and function of FOXM1 [35, 36, 37, 38, 39], a master regulator of cell cycle progression. FOXM1 protein levels also dynamically change with cell cycle progression [40, 41], whereas the functional activation, nuclear translocation, and stability of FOXM1 protein are subject to its protein phosphorylation, regulated by cyclin/CDK and KRAS‐MAPK/AKT signaling [39, 42, 43]. FOXM1 is involved in promoting cell proliferation and facilitating tumor cell invasion, metastasis, and stemness [20, 44, 45, 46] and elevated expression of FOXM1 protein is correlated with various human malignancies, including pancreatic cancer. FOXM1 was shown to be a major predictor of adverse outcomes among 39 human tumor types [20, 35, 47, 48, 49, 50, 51]. Altogether, these data establish FOXM1 as a promising target for cancer treatment [52] and FOXM1 inhibitors are being developed for clinical application [53, 54].

The current study is the first to show that BT can significantly affect protein expression of FOXM1 and its related signaling loop genes, including ALKBH5, cyclin B1, and CDK1, in PANC‐1 cells, in a blinded RPPA analysis. Knockout of the FOXM1 gene blocked the effects of BT on the G0/G1 phase of cells and on cell invasion, whereas overexpression of FOXM1 augmented the activity of BT, which substantiates the critical role of FOXM1 in mediating the antitumor activity of BT. Detailed mechanisms underlying how BT regulates FOXM1 expression and function remain to be defined. FOXM1 expression and activity are tightly regulated and coordinated by transcriptional factors (activation) and epigenetic modifications. Human AlkB homolog H5 (ALKBH5), an important m6A demethylase that plays an important role in proliferation and metastasis during the development of various neoplastic conditions, has been reported to demethylate nascent FOXM1 transcripts and enhance FOXM1 expression in glioblastoma. Given that BT administered for only a short time (15–30 min) can result in significant downregulation of FOXM1 protein expression, it is possible that posttranscriptional modifications at the FOXM1 mRNA and/or protein level, such as ALKBH5‐mediated m6A methylation of FOXM1 mRNA or pAKT‐mediated phosphorylation of FOXM1 protein, may contribute to the observed changes in FOXM1 protein expression after BT [55, 56]. Emerging evidence suggests that FOXM1 may reciprocally promote AKT activation and establish a positive feedback loop [52, 55]. Our previous study showed that pAKT can be downregulated by BT treatment [15]. Thus, BT might potentially affect the network of the FOXM1 and AKT pathway in PDAC cells. Given short‐term exposures to radiation (x‐ray, 10–15 min) result in measurable changes in DNA methylation or epigenetic changes in *Candida albicans* [57], exposure to short‐term energy might be able to alter epigenetic modifications such as methylation. Whether BT‐induced FOXM1 suppression after 15 min treatment is due to methylation changes of the cells via external mechanisms (heating/lighting) deserves further investigation.

It is well appreciated that cancer cells possess distinct bioelectrical properties. Abnormal depolarization of resting membrane *V*_*m*_ has been considered a convenient marker for neoplasia and activates a metastatic phenotype in genetically normal cells in vivo [58, 59, 60, 61]. *V*_*m*_ change triggers metastatic behaviors at a considerable distance, mediated by transcriptional and epigenetic effects of electrically modulated flows of serotonin and butyrate [58]. Cell membrane voltage potential is mediated through different concentrations of ions and ion channels that are now recognized to play an important role in oncogenesis [61]. Hyperpolarizing CHO cells induce mitotic arrest and cell division, and hyperpolarization inhibits oncogene‐induced tumorigenesis [31, 62]. Using a well‐characterized molecule, DiBAC4, for staining the cell membrane voltage potential, we observed that BT significantly reduced the cell membrane voltage potential in PANC‐1 and MiaPaCa‐2 (human), and KPCY (murine) cells. It is well established that cell migration is regulated by water and ions [63], and *V*_*m*_ is considered an indirect factor that can affect cell migration by regulating the electrical driving force for Ca^2^+ via the Transient Receptor Potential (TRP) channel [63, 64]. Several members of the TRP channel, such as TRPM8, are critical in the proliferation and metastasis of pancreatic cancer cells, and expression of TRPM8 is associated with poor overall survival of patients with PDAC [65, 66, 67, 68]. Thus, how BT led to the reduction of cell *V*_*m*_, resulting in hyperpolarization of PDAC cells and contributing to the antimetastatic activity of PDAC cells, needs to be further evaluated.

The physical mechanisms by which BT affects cellular processes remain unknown. Potential mechanisms may include biophoton emissions or electromagnetic fields (EMFs) [69, 70, 71, 72, 73], although these were not directly assessed in this study. The human body is well known to emit EMFs, which are intricately involved in maintaining homeostasis overall and in many specific organ systems. Electrocardiograms and EEGs are used to measure the EMFs emitted by the heart and brain, respectively, and other technologies can be used to measure human EMFs, such as examination of the emission of biophotons from the body [74]. EMFs can also be measured in any living organism [75]. EMFs have been shown to modulate tumor growth, angiogenesis, and tumor necrosis factor in animals [76], including inhibiting EpH4‐MEK‐Bcl2 breast tumor growth in mice via induction of apoptosis [77]. EMFs have started to be used in conventional cancer treatment. For example, the US Food and Drug Administration approved the use of low frequency alternating electric fields for the treatment of refractory glioblastoma multiforme (known as tumor treating fields) and other cancers including PDAC [78, 79, 80].

A limitation of the current research is that we did not directly measure or manipulate any of the purported mechanisms associated with BT, such as EMFs, biophoton emissions, or other mechanisms of action. Ongoing and planned studies include incorporating techniques to block EMF, assessing biometrics of the therapists including EEG and EKG, measuring changes in the immediate environment, and exploring the potential role of quantum physics properties [81, 82]. Further in‐depth research will help to reveal how BT may induce alterations in cell biology and transcriptional control. Another limitation in these series of experiments was that while we did include a SC group in all experiments, which involved a non‐therapist human moving in a similar manner to mimic the movements of the biofield therapists, they were not instructed to engage in any specific visualizations. However, the SC accounted for the presence of a human and the effects of passage of time on the cells/mice in the laboratory environment on the bench. Although the study had the therapists use the Bengston Method, it is unclear the extent to which they engaged in cycling, as we cannot measure the degree to which they were performing the Bengston Method. We also cannot know if the effects we found are specific to this technique, as we did not study other BT techniques. Future research should examine additional BT modalities for which people can be trained such as Healing Touch, Reiki, Therapeutic Touch, or other techniques. It may be that different techniques work well for some conditions and not others.

Conducting laboratory‐based BT research presents unique methodological challenges. Laboratory environments contain numerous instruments that generate electromagnetic interference, potentially influencing experimental outcomes. Such interference could originate from equipment in the immediate setting or even on adjacent floors. To minimize bias and maintain consistency, it is critical that the SCs are exposed to the same laboratory conditions as the BT group. Timing of interventions is another important consideration, particularly in animal studies where circadian rhythms may affect results. Ensuring consistency in the time of day for both BT and sham conditions helps reduce variability, as done in these studies. Blinding the laboratory staff who conduct the assays to group assignments is essential to prevent inadvertent bias. Measures must also be taken to avoid cross‐contamination between BT and sham conditions. This includes housing cells in separate incubators, maintaining animals in different areas of the colony, and thoroughly cleaning treatment locations between sessions. These precautions help preserve the integrity of experimental conditions and strengthen the validity of findings.

In conclusion, although both human and preclinical studies suggest that BT may have a role in cancer treatment, there is a lack of systematic evaluation to fully understand the effect of BT on cancer cell proliferation and metastasis, especially in pancreatic cancer. Our data suggest that the BT technique used in these series of experiments exerted antitumor activity against pancreatic cancer cells and PDOs in culture and their relevant orthotopic xenograft models. The in vitro studies suggest that BT modulates important cell cycle regulators such as FOXM1 and p21 and results in cell cycle arrest and cell hyperpolarization. Furthermore, we showed for the first time that BT treatment alters mitochondria ultrastructure and has the potential to reduce metastasis of pancreatic cancer cells. Understanding the mechanisms responsible for the antimetastatic activity of BT warrants further investigation.

## Author Contributions

**Peiying Yang:** conceptualization, methodology, formal analysis, supervision, funding acquisition, project administration, writing – original draft, writing – review and editing. **Daoyan Wei:** conceptualization, methodology, project administration, writing – original draft, writing – review and editing. **Sharmistha Chakraborty:** methodology, investigation, formal analysis, writing – original draft, writing – review and editing. **Phuong Nguyen:** methodology, investigation, formal analysis, project administration, writing – original draft, writing – review and editing. **Andrew Cusimano:** investigation, writing – review and editing. **Defeng Deng:** methodology, investigation, formal analysis, writing – original draft, writing – review and editing. **Shafaqmuhammad Iqbal:** investigation, formal analysis, writing – review and editing. **Monica Nelson:** investigation, writing – review and editing. **Meng Cui:** methodology, investigation, writing – review and editing. **Jianliang Dai:** data curation, formal analysis, writing – review and editing. **Mihai Gagea:** validation, formal analysis, writing – review and editing. **Richard Wagner:** investigation, writing – review and editing. **Yisheng Li:** validation, formal analysis, writing – review and editing. **Lorenzo Cohen:** conceptualization, methodology, supervision, funding acquisition, project administration, writing – original draft, writing – review and editing.

## Funding

Support for this research was provided by the Emerald Gate Charitable Trust and by core facility support through National Cancer Institute grant P30 CA016672 to The University of Texas MD Anderson Cancer Center.

## Disclosure

Trial registration: Exempt from trial registration (MD Anderson Cancer Center IRB Protocol 2020–1210).

## Conflicts of Interest

The authors declare no conflicts of interest.

## Supporting information


**Figures S1‐S13:** cam471726‐sup‐0001‐FigureS1‐S13.pdf.

## Data Availability

Data is provided within the manuscript or Supporting Information files. The protocol was preregistered with Open Science Framework https://osf.io/6cx3b/overview.
