# Supplementary material for: The Preclinical Effects and Mechanisms of Biofield Therapy on Pancreatic Cancer Cell Growth and Metastasis
Source: Cancer Med. 2026 Apr 13;15(4):e71726. doi: 10.1002/cam4.71726 (PMC13071467; doi:10.1002/cam4.71726)
Supplement: Supplementary file 1 — Figures S1‐S13: cam471726‐sup‐0001‐FigureS1‐S13.pdf. [file CAM4-15-e71726-s001.pdf]

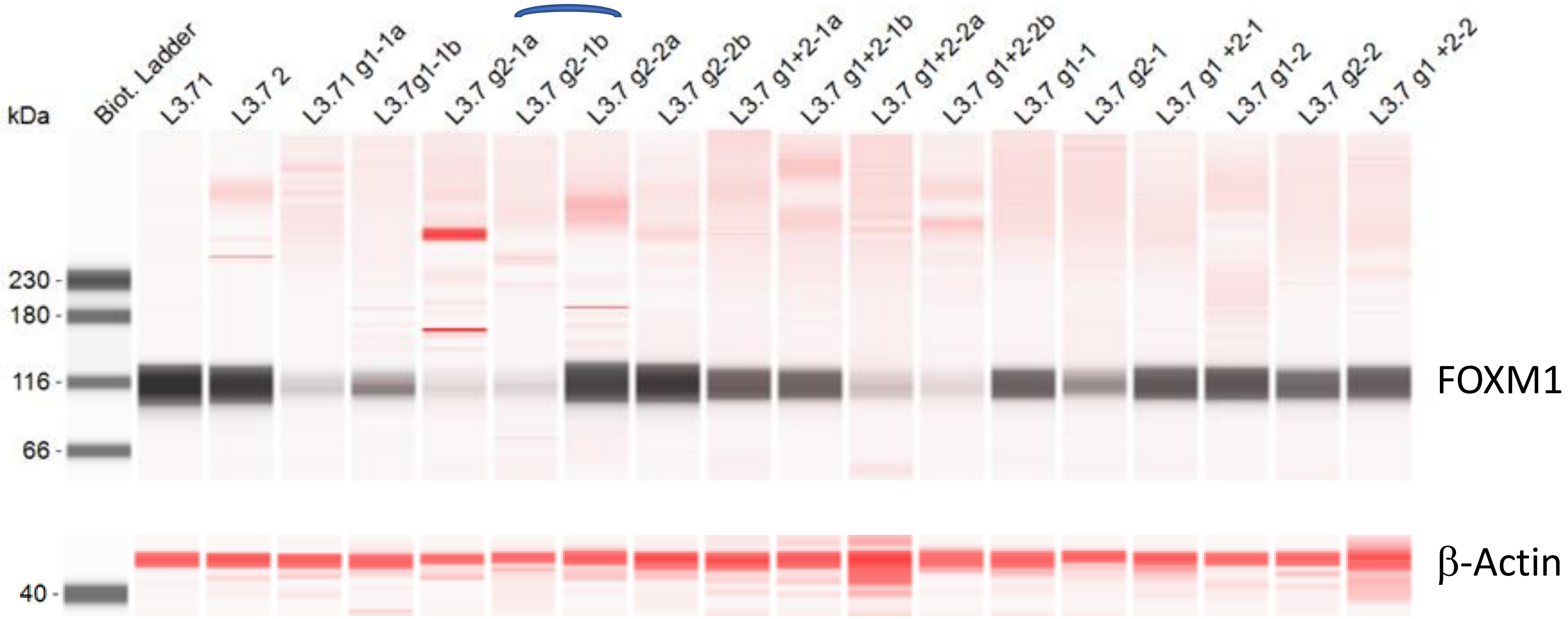

Supplemental Figure S1. FOXM1 protein expression in L3.7 wild-type and L3.7 CRISPR-Cas9 FOXM1 knockdown clones, examined by Jess.

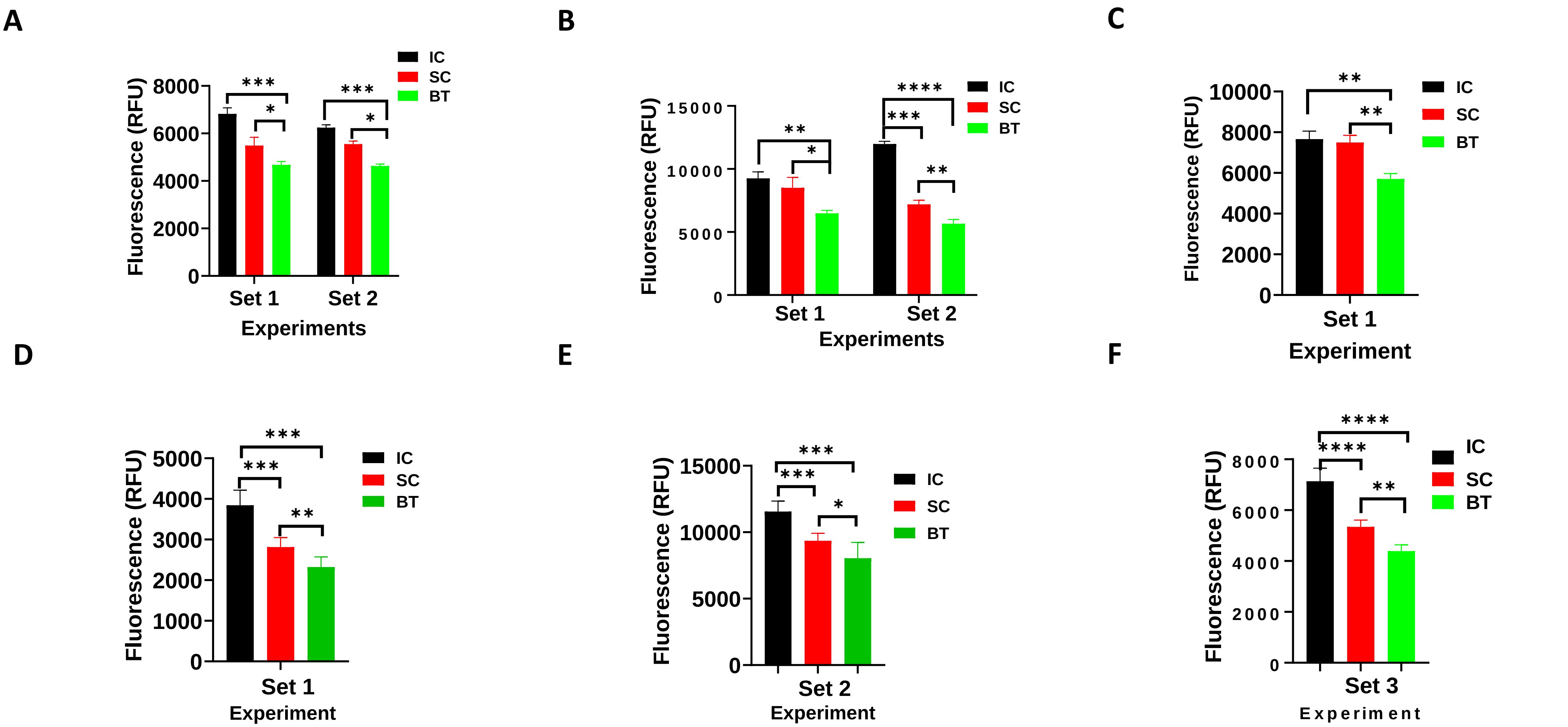

Supplemental Figure S2. Effect of biofield therapy (BT), at 1 hour after 15 minutes (PANC-1 cells) or 30 minutes (Panc02 cells) of treatment, on the growth of PANC-1 cells (A-C) in five different sets of experiments and Panc02 cells (D-F) in three different sets of experiments, compared with sham control (SC) and incubator control (IC). Data are presented as mean±SD (*p<0.05; **p<0.01; ***p<0.001; ****p<0.0001; n>4).

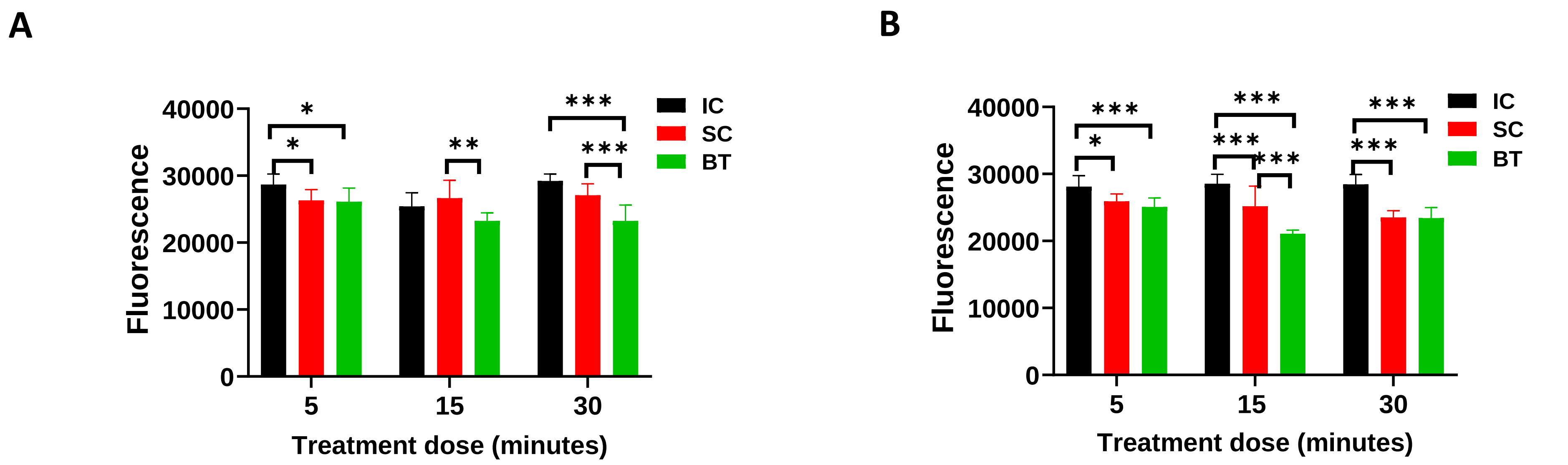

Supplemental Figure S3. Effect of biofield therapy (BT), compared with sham control (SC) and incubator control (IC), on the growth of human pancreatic cancer MiaPaCa-2 (A) and mouse pancreatic cancer KPCY cells (B). Data are presented as mean±SD (*p<0.05; **p<0.01; ***p<0.001; n>4).

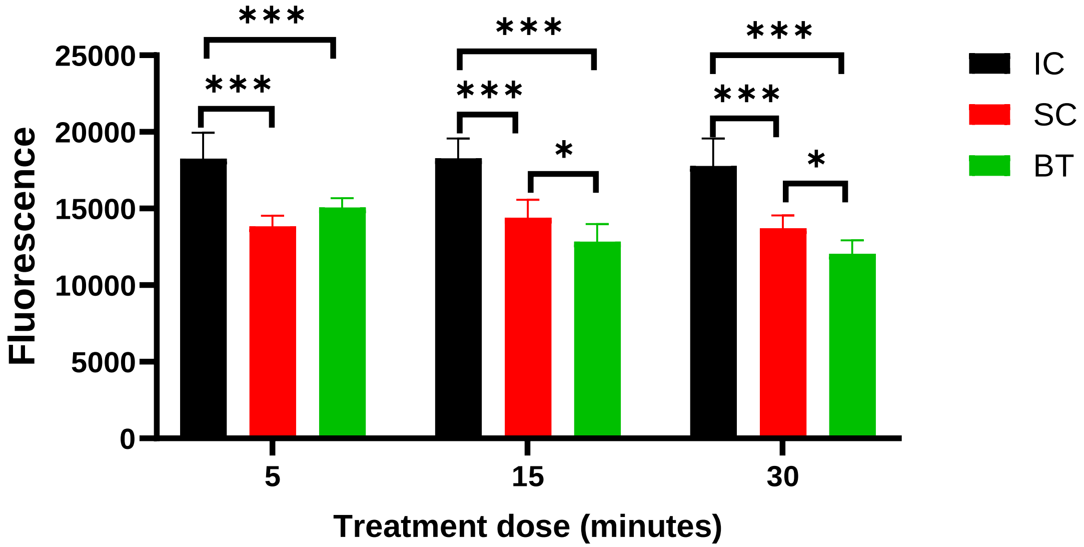

Supplemental Figure S4. Effect of biofield therapy (BT), compared with sham control (SC) and incubator control (IC), on the growth of PANC-1 cells treated by Therapist 3. Data are presented as mean±SD (*p<0.05; ***p<0.001; n>4).

Supplemental Figure 5A

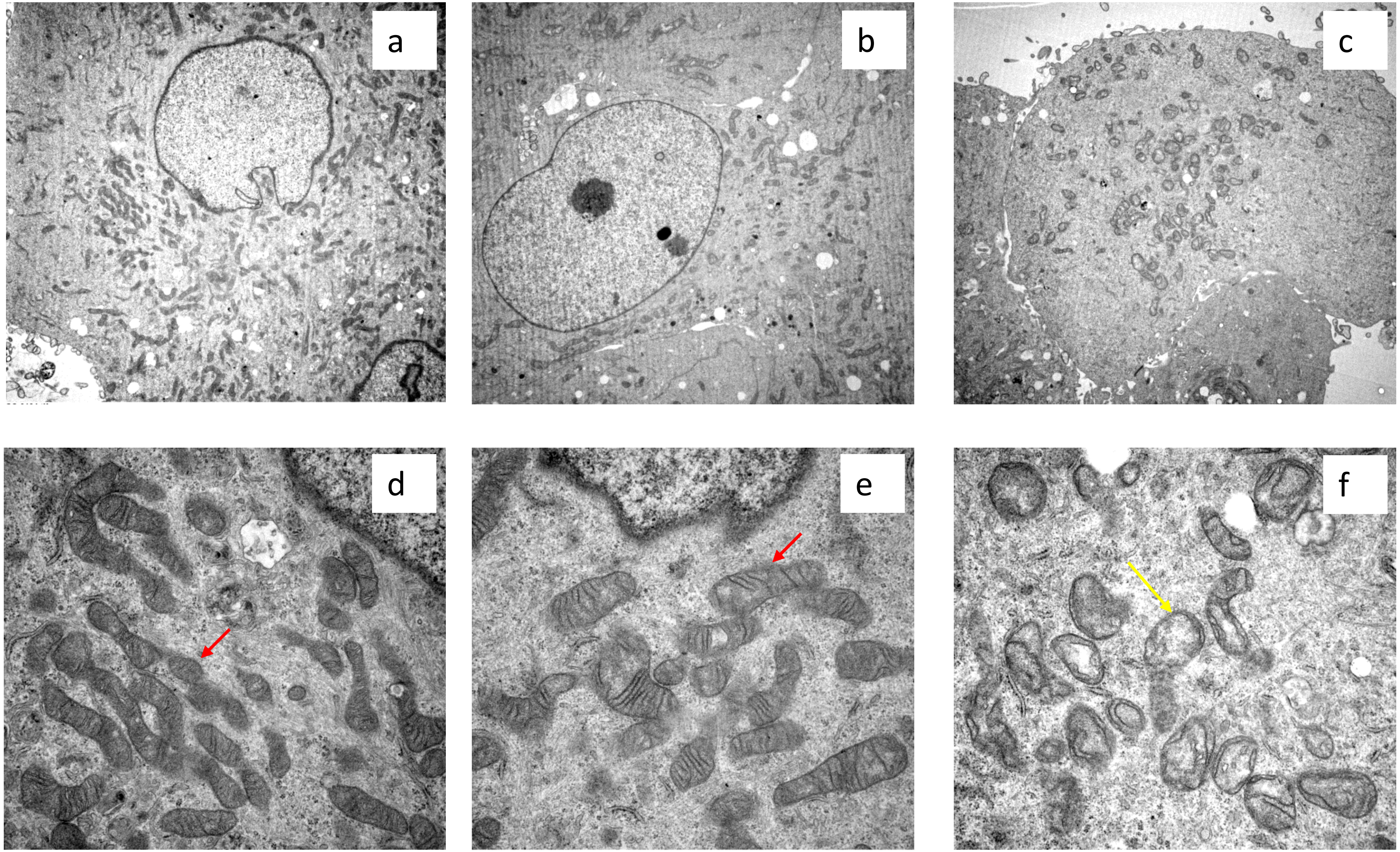

Supplemental Figure 5B

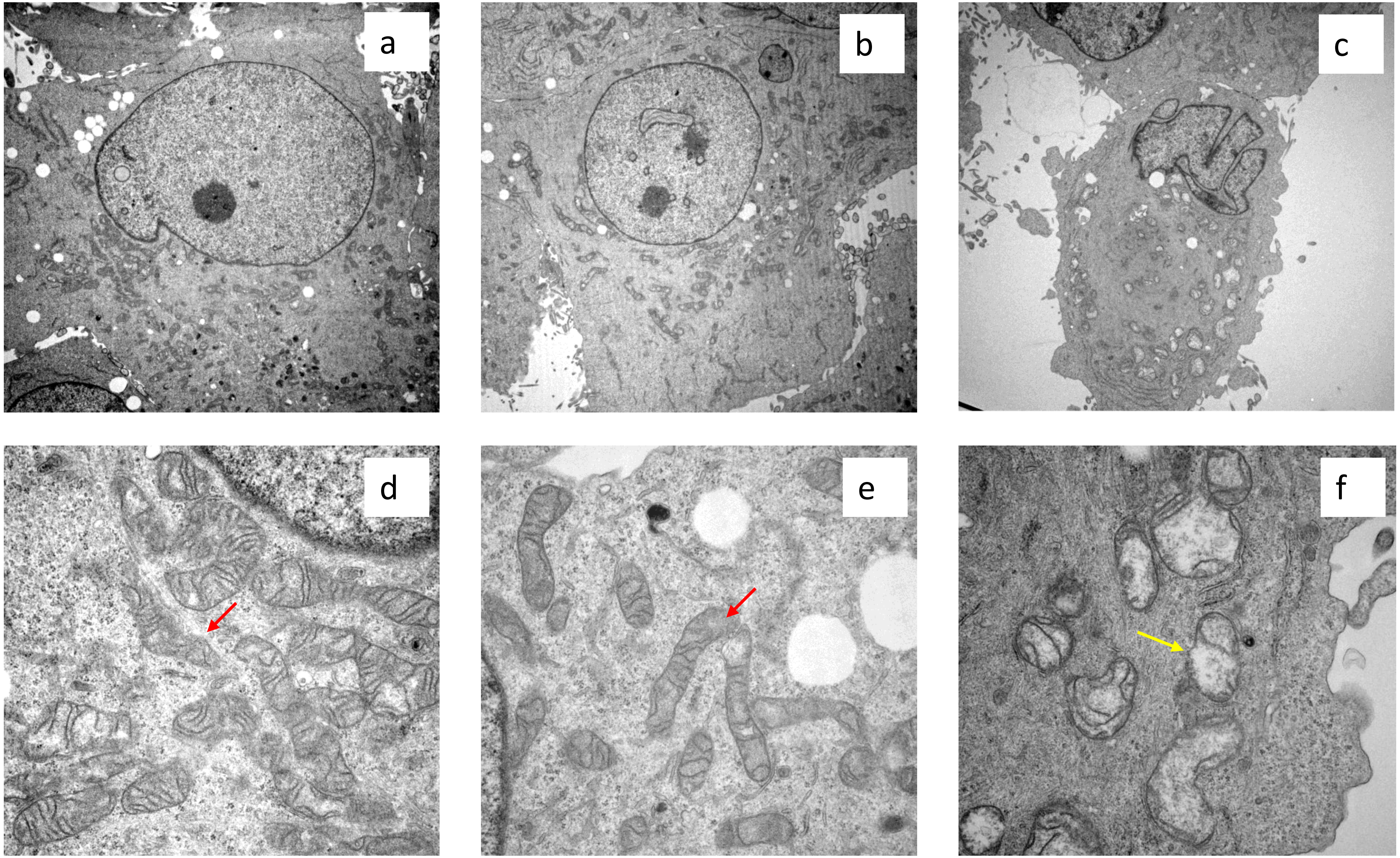

Supplemental Figure S5. Transmission electron micrographs showing morphologic changes in PANC-1 cells in the incubator control (IC) group (a and d), sham control (SC) group (b and e), and biofield therapy (BT) group (c and f) after 15 (A) and 30 minutes (B) of exposure to treatment (samples were collected immediately after treatment). Note the normal appearance mitochondria (red arrows) in the IC and SC groups (d and e) and swollen mitochondria in the BT group (yellow arrow, f). Images a-c were taken at 5000×; images d-f were taken at 25,000×.

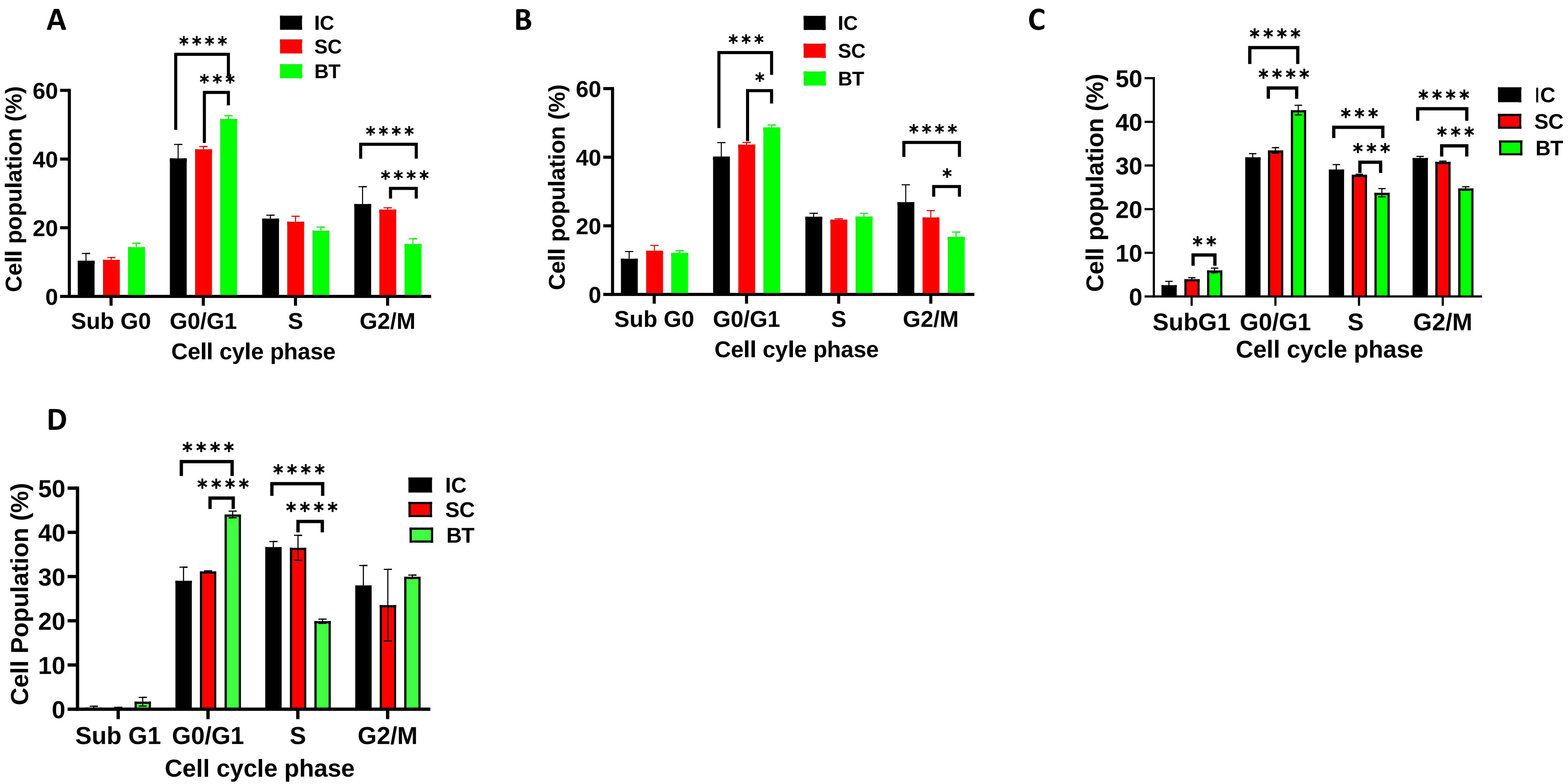

Supplemental Figure S6. (A-B) Effect of biofield therapy (BT), sham control (SC), or incubator control (IC) on the cell cycle of PANC-1 cells in another two separate studies. (C-D) Cell cycle of PANC-1 cells after 15-minute exposure to BT provided by Therapist 2 (C) and Therapist 3 (D). Data are presented as mean±SD (*p<0.05; **p<0.01; ***p<0.001; ****p<0.0001; n>4).

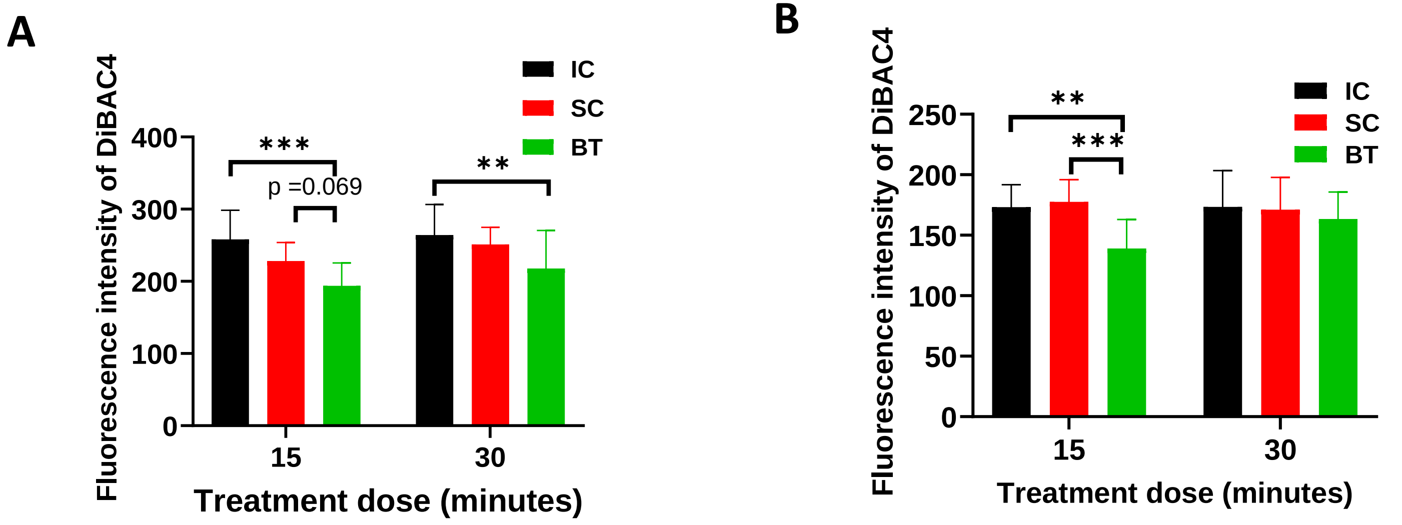

Supplemental Figure S7. Cell membrane voltage potential of MiaPaCa-2 (A) and KPCY (B) cells measured immediately after biofield therapy (BT), sham control (SC), or incubator control (IC). Data are presented as mean±SD (**$p<0.01$; ***$p<0.001$; $n>6$).

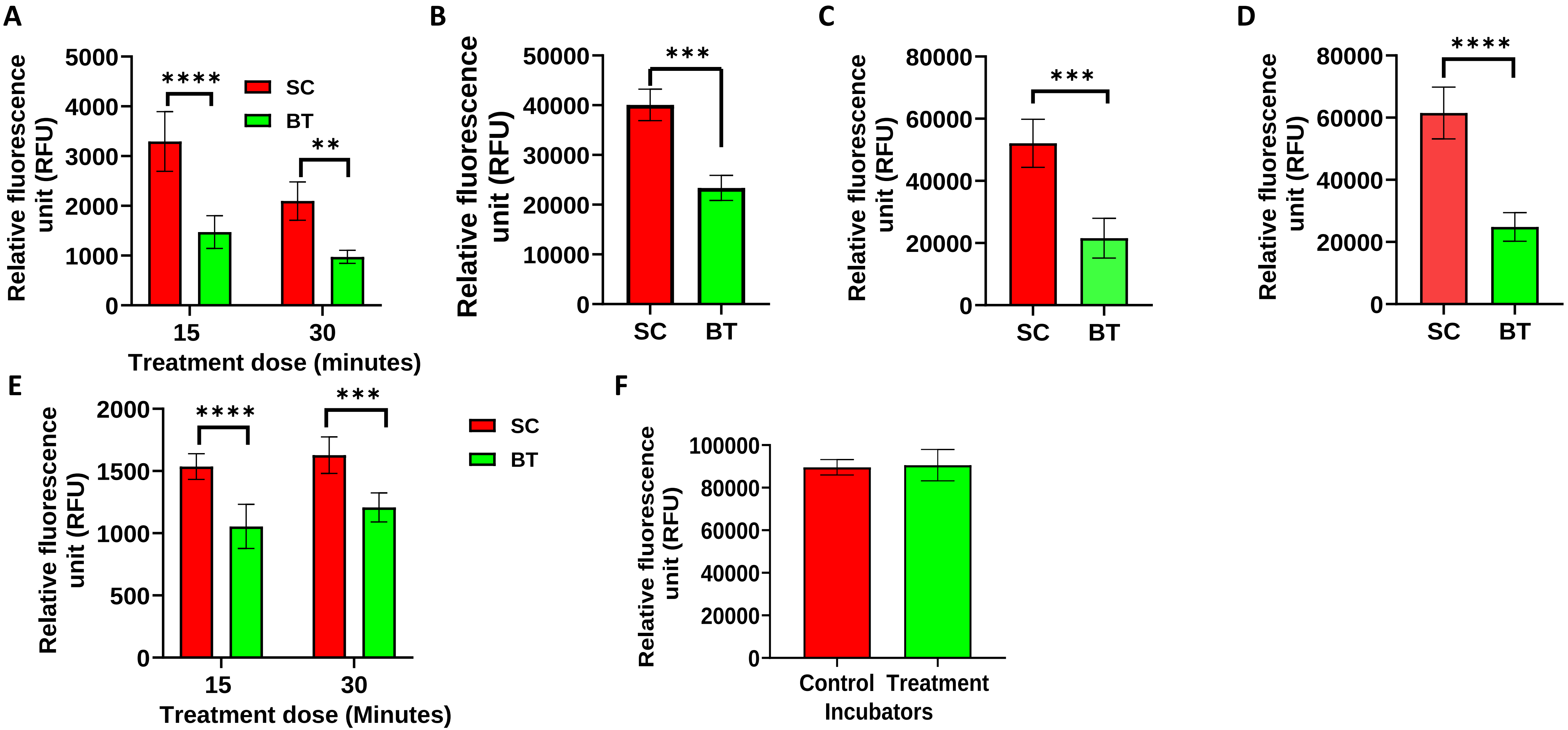

Supplemental Figure S8. Biofield therapy (BT) significantly inhibited the invasiveness of PANC-1 cells (A-D) and L3.7 cells (E) after they were treated by Therapist 1 for 15 or 30 minutes, compared with sham control (SC). (F) Invasiveness of PANC-1 cells were tested after they were incubated in the SC or BT incubator for 48 hrs. Data are presented as mean±SD (**p<0.01; ***p<0.001; ****p<0.0001).

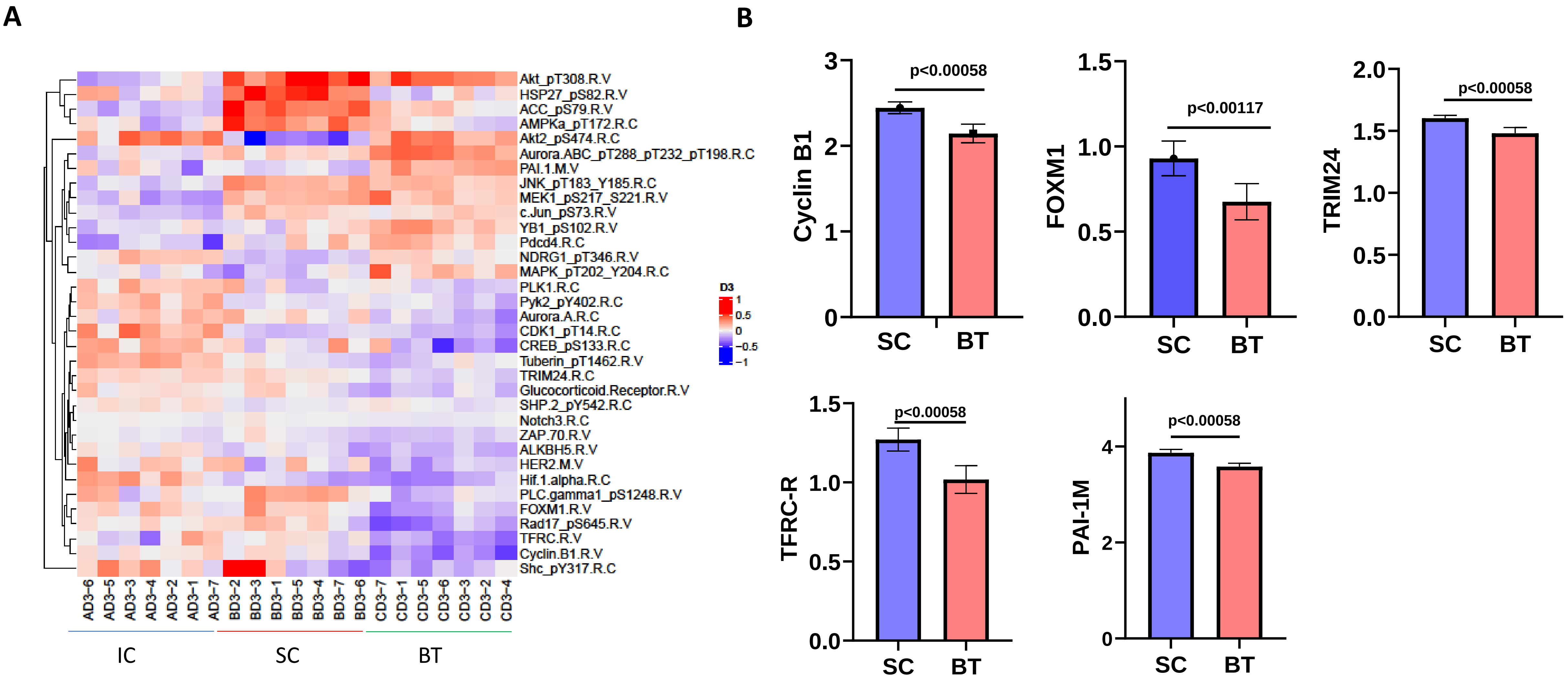

Supplemental Figure S9. Expression of proteins that regulate the cell cycle and cell signaling in PANC-1 cells exposed to biofield therapy (BT) for 30 minutes, compared with sham control (SC). (A) Heatmap of cell cycle regulating proteins and cell signaling proteins in PANC-1 cells, as examined by reverse phase protein array (RPPA). The proteins included in this heatmap were statistically significant among different treatment groups according to Wilcoxon rank-sum analysis, with p values lower than the Benjamini-Hochberg thresholds for false discovery rate (<0.05). (B) Expression of cell cycle regulating proteins and epigenetic modification proteins such as cyclin B1, FOXM1, and TRIM24 in PANC-1 cells exposed to BT, compared with SC, as examined by RPPA (n=7).

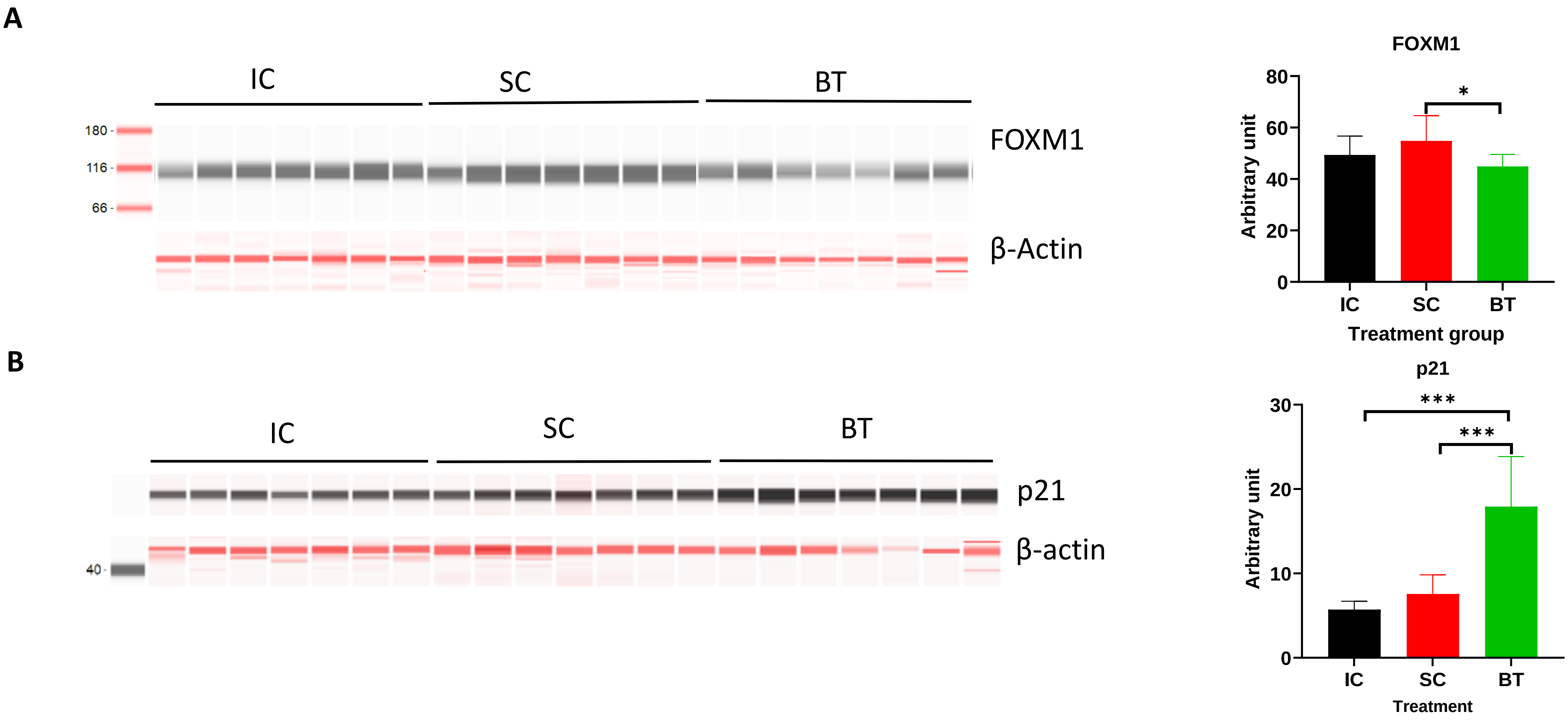

Supplemental Figure S10. Expression of FOXM1 (A) and p21 (B) proteins in L3.7 cells exposed to biofield therapy (BT) for 15 min compared with sham control (SC) and incubator control (IC), examined by Jess. Data are presented as mean±SD (*p<0.05; ***p<0.001).

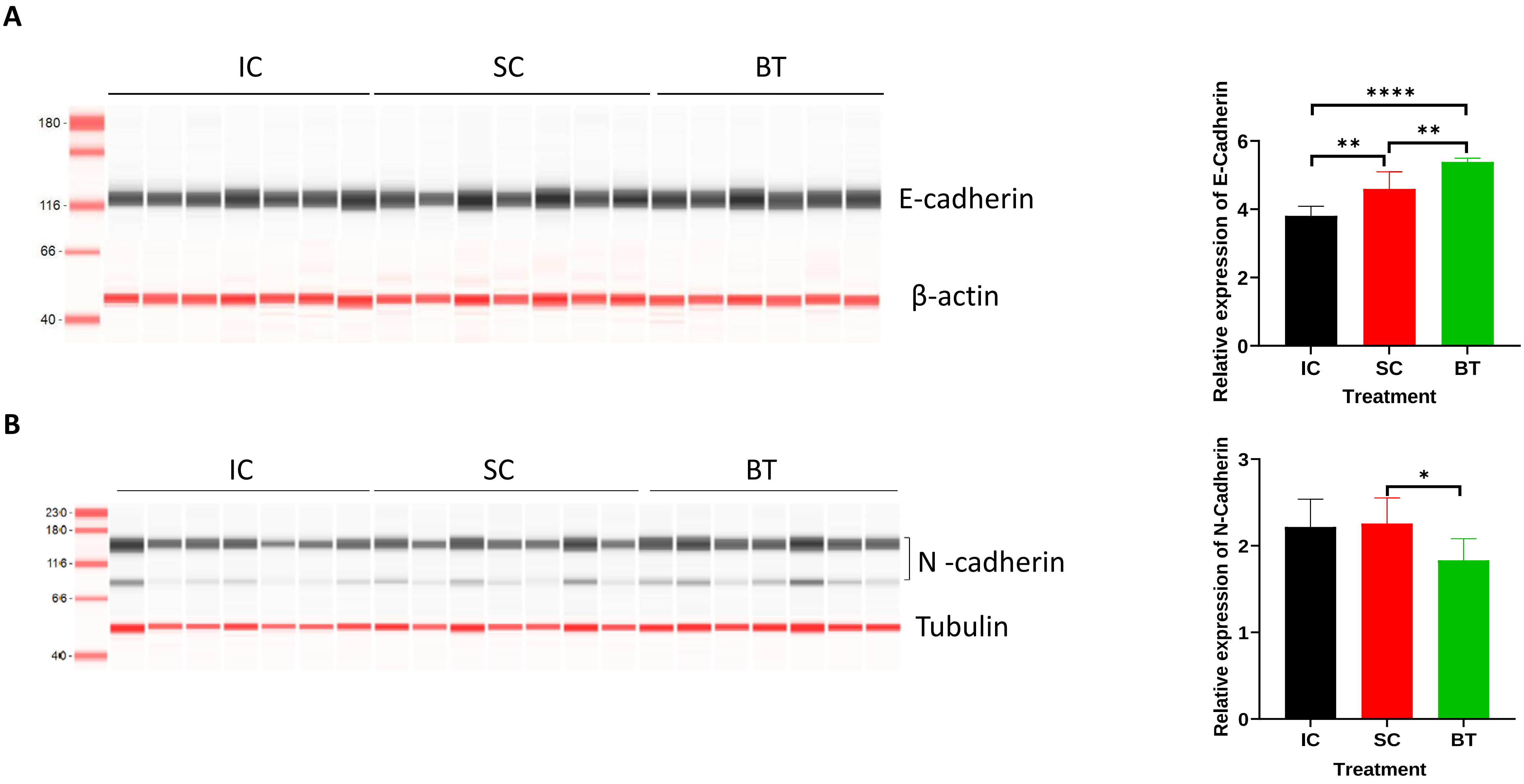

Supplemental Figure S11. Effect of biofield therapy (BT), compared with sham control (SC) and incubator control (IC), on epithelial-mesenchymal transition in PANC-1 cells. Western blot analysis of E-cadherin (A) and N-cadherin (B) was conducted in PANC-1 cells 24 hrs after 15-minute BT. Data are presented as mean±SD (*p<0.05; **p<0.01; ****p<0.0001).

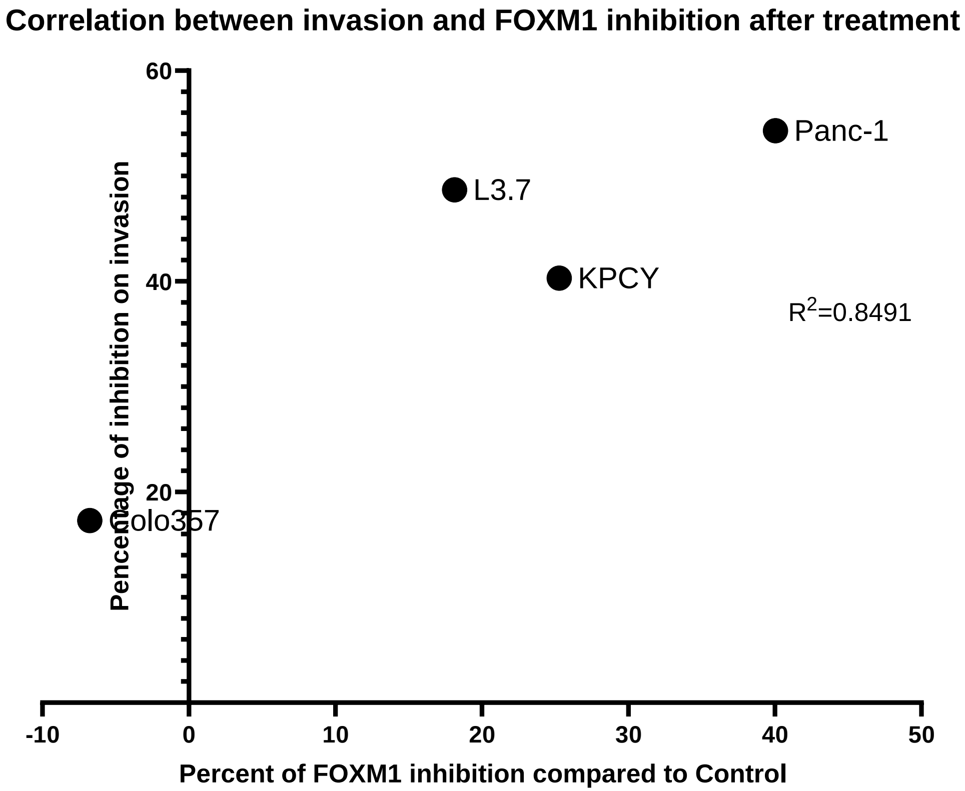

Supplemental Figure S12. Correlation between the percentage of FOXM1 inhibition induced by BT and the percentage inhibition of PDAC cell invasiveness by BT.

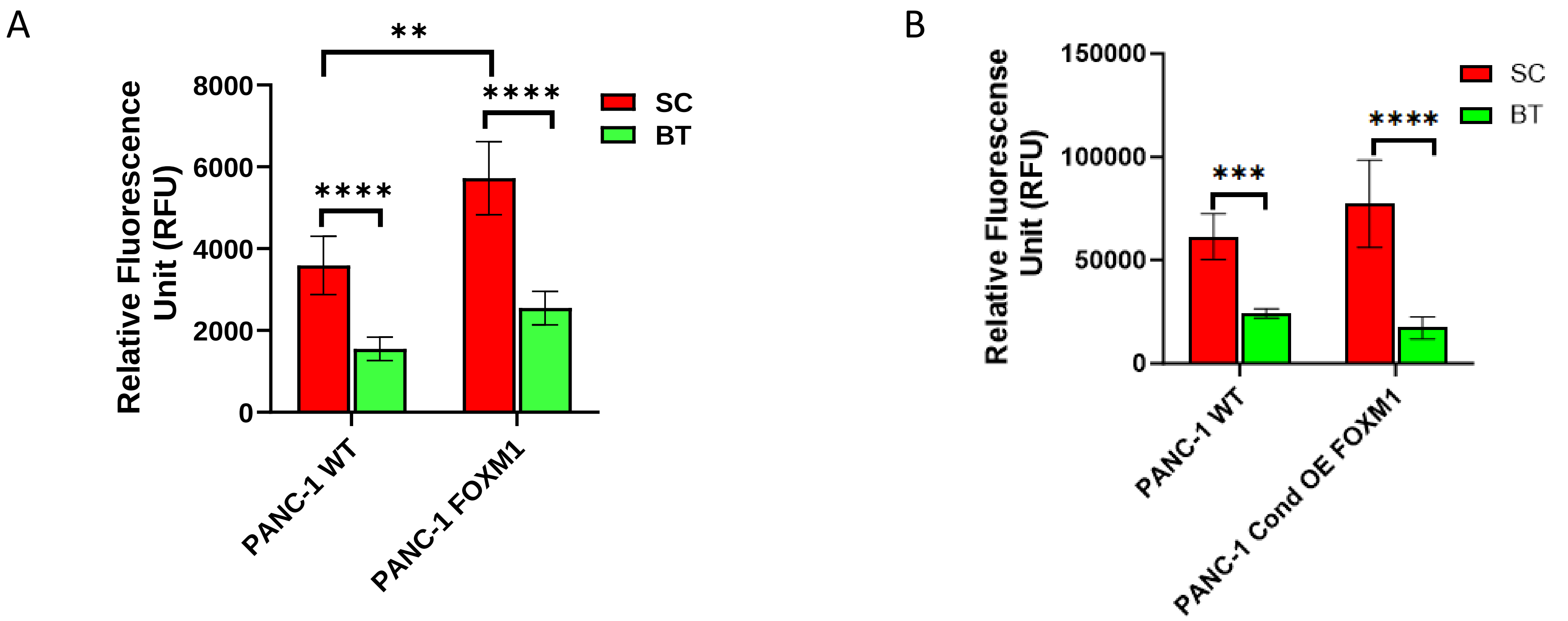

Supplemental Figure S13. Effect of FOXM1 overexpression on biofield therapy (BT) elicited anti-invasiveness in PANC-1 cells. (A) Invasiveness of control PANC-1 and FOXM1 stably overexpressing PANC-1 cells exposed to sham control (SC) or BT. (B) Invasiveness of control PANC-1 and Tet-inducible FOXM1 overexpressing PANC-1 cells exposed to SC or BT. Data are presented as mean±SD (** $p < 0.01$, *** $p < 0.001$, **** $p < 0.0001$).
